# In Vitro Study of Cytotoxic Mechanisms of Alkylphospholipids and Alkyltriazoles in Acute Lymphoblastic Leukemia Models

**DOI:** 10.3390/molecules27238633

**Published:** 2022-12-06

**Authors:** Larissa de Oliveira Passos Jesus, Aline Aparecida de Souza, Heron Fernandes Vieira Torquato, Vanessa Silva Gontijo, Rossimirian Pereira de Freitas, Tarsis Ferreira Gesteira, Vivien Jane Coulson-Thomas, Ricardo José Soares Torquato, Aparecida Sadae Tanaka, Edgar Julian Paredes-Gamero, Wagner Alves de Souza Judice

**Affiliations:** 1 Interdisciplinary Center for Biochemical Research, University of Mogi das Cruzes (UMC), Mogi das Cruzes 08780-911, SP, Brazil; 2 Faculty of Pharmacy, Centro Universitário Braz Cubas (UBC), Mogi das Cruzes 08773-380, SP, Brazil; 3 Faculty of Pharmaceutical Sciences, Food and Nutrition, Federal University of Mato Grosso do Sul (UFMS), Campo Grande 79070-900, MS, Brazil; 4 Phytochemistry and Medicinal Chemistry Laboratory (LFQM), Institute of Chemistry, Federal University of Alfenas (UNIFAL), Alfenas 37130-000, MG, Brazil; 5 Institute of Exact Sciences, Department of Chemistry, Federal University of Minas Gerais (UFMG), Belo Horizonte 31270-901, MG, Brazil; 6 College of Optometry, University of Houston, Houston, TX 77204, USA; 7 Department of Biochemistry, Federal University of São Paulo, São Paulo (UNIFESP), São Paulo 04044-020, SP, Brazil

**Keywords:** alkylphospholipids, alkyltriazoles, cell death, acute lymphoblastic leukemia, cathepsin inhibition

## Abstract

This study investigates the efficacy of miltefosine, alkylphospholipid, and alkyltriazolederivative compounds against leukemia lineages. The cytotoxic effects and cellular and molecular mechanisms of the compounds were investigated. The inhibitory potential and mechanism of inhibition of cathepsins B and L, molecular docking simulation, molecular dynamics and binding free energy evaluation were performed to determine the interaction of cathepsins and compounds. Among the 21 compounds tested, **C9** and **C21** mainly showed cytotoxic effects in Jurkat and CCRF-CEM cells, two human acute lymphoblastic leukemia (ALL) lineages. Activation of induced cell death by **C9** and **C21** with apoptotic and necrosis-like characteristics was observed, including an increase in annexin-V^+^propidium iodide^−^, annexin-V^+^propidium iodide^+^, cleaved caspase 3 and PARP, cytochrome c release, and nuclear alterations. Bax inhibitor, Z-VAD-FMK, pepstatin, and necrostatin partially reduced cell death, suggesting that involvement of the caspase-dependent and -independent mechanisms is related to cell type. Compounds **C9** and **C21** inhibited cathepsin L by a noncompetitive mechanism, and cathepsin B by a competitive and noncompetitive mechanism, respectively. Complexes cathepsin-**C9** and cathepsin-**C21** exhibited significant hydrophobic interactions, water bridges, and hydrogen bonds. In conclusion, alkyltriazoles present cytotoxic activity against acute lymphoblastic lineages and represent a promising scaffold for the development of molecules for this application.

## 1. Introduction

**Figure 1:**
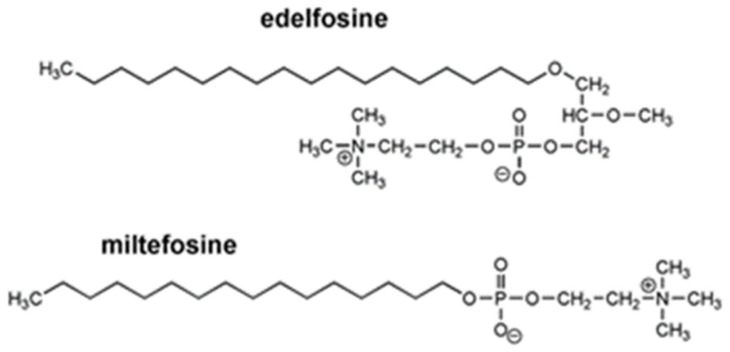
Molecular structures of edelfosine and miltefosine. Edelfosine (2-methoxy-3-(octadecyloxy)propyl 2-(trimethylazaniumyl)ethyl phophate) is a synthetic alkylphospholipid (APL). Miltefosine (hexadecyl 2-(trimethylazaniumyl)ethyl phosphate).

The unspecific activity of most of the antitumor compounds and their side effects have encouraged the research on new synthetized molecules. Antitumor alkylphospholipids (APLs) do not target cellular DNA or cytoskeletons, as is the case for the typical antitumor medicaments cisplatin or taxol, but instead are involved in cell membrane turnover and regulation of cellular physiology [1,2]. For instance, edelfosine (1-*O*-octadecyl-2-*O*-methyl-sn-glycero-3-phosphocholine; ET-18–OCH_3_) and miltefosine (hexadecylphosphocholine, HePC) are prototype antitumor APL molecules [3,4]. Edelfosine has been shown to be effective in the treatment of acute leukemia by the induction of apoptosis, thus making it a template of new ether derivatives [5]. Edelfosine induces caspase- and mitochondrial-mediated apoptosis in pancreatic cancer cells via stress responses, with participation of the endoplasmic reticulum and caspase-4 and c-Jun (the name Jun comes from the Japanese translation of the number 17 “ju-nana”) NH_2_-terminal kinase activation [6]. A study performed using cholesterol monolayers that mimic normal and tumor cell membranes showed that edelfosine enters tumor cellular membranes more easily than healthy membranes [7]. Similarly, studies have demonstrated that miltefosine presents antitumor activity in vitro; however, its clinical use is restricted to topical application since it possesses strong hemolytic activity [8]. For these reasons, edelfosine and miltefosine are considered as useful structural models in the search for new APLs (Figure 1).

Edelfosine has two different subcellular locations, plasma membrane lipid rafts and the endoplasmic reticulum, which depend on the cell type. Edelfosine can affect two distinct targets, leading to apoptosis by different signaling mechanisms in human leukemia and solid tumor cells [9]. In the leukemia cells, the concentration of edelfosine in membrane rafts will promote co-clustering of lipid rafts with Fas receptor and downstream signaling molecules, leading to a rapid apoptotic response [10].

The anticancer activity of miltefosine involves inhibition of phosphatidylcholine biosynthesis and induction of apoptosis by inhibiting the PI3K/Akt/PKB pathway. These mechanisms cause intracellular stress by blocking signals that are essential for survival or by inducing various pro-apoptotic cell signaling pathways [11]. No adverse effects under general conditions or any treatment-related macroscopic changes in major organs, including the brain, liver, and kidneys, were induced by this therapeutically effective regimen. No symptoms associated with central nervous system disorders have been noted in patients, to date, with the exception of manageable toxicities, such as anemia, diarrhea, and nausea [12,13].

Erufosine offers potential as a novel therapeutic for cancer with a reduced toxicity profile to bone marrow cells, compared with other agents in this class. The main adverse effect of edelfosine and miltefosine is gastrointestinal toxicity [14]. On the other hand, intravenous and oral treatment with edelfosine has been used only for bone-marrow purging in patients with acute leukemia [15]. Studies that combine APL with other drugs have shown that such a therapeutic approach can result in numerous clinical benefits [16].

In addition, a new series of analogs with simple structures that contain a long alkyltriazoles chain have been synthesized and their effects evaluated in tumor cells [17]. The 1,2,3-triazole ring acts not only as a pharmacophore, but also as a linker between two or more substances through a molecular hybridization strategy. The triazole ring is stable, since it does not undergo hydrolysis, oxidation or reduction. Triazole can also act as an amide group bioisostere, exhibiting similar physicochemical properties. The triazole unit acts as a rigid binder and can accommodate two alkyl groups [18]. Studies have reported 1,2,3-triazole derivatives with bactericidal [19] and anticancer [20] activity. These derivatives can induce cell cycle arrest and apoptosis of cancer cells [13,21].

1,2,3-triazole derivatives showed promising anticancer effects on T-acute lymphoblastic leukemia cell lines (CCRF-CEM), inhibited the growth of SR leukemia cell lines; presented cytotoxicity against HL-60 (acute promyelocytic leukemia) and Jurkat (acute T cell leukemia) cells [22]. Various studies have evaluated the effects of cytotoxic activity of alkyl-triazole derivatives on MCF-7 and MDA-MB-231 breast cancer cells [23], THP-1 (HUMAN acute monocytic leukemia), Colo205 (colon cancer), U937 (human myeloid leukemia) and HeLa (human cervical carcinoma) cells [24]. Derivative compounds of 1,2,3-triazoles have shown anticancer potential through the inhibition of different enzymes, such as EGFR (epidermal growth factor), VEGFR (vascular endothelial growth factor) and PARP (poly (ADP-ribose) polymerase), which are involved in the progression of this devastating disease [22].

Adverse effects such as rash, diarrhea, headache, hepatotoxicity, and gastrointestinal problems, including several severe problems, are reported for many triazole drugs [25]. On the other hand, *S*-Alkyl-1,2,4-triazoles showed toxic effects against *T. cruzi* with low toxicity to host cells [26].

Lymphomas and leukemias are cancers mainly produced by molecular alterations in blood and bone marrow cells. Among them, acute lymphocytic leukemia (ALL) is the most common type of cancer in children, but can also occur in adults [27]. Adult patients with ALL are often unable to tolerate the side effects of the required intensive chemotherapy treatment. In contrast, targeted therapies, such as Notch inhibitors, FMS-related targeted kinase-3, proteasome inhibitors and hypomethylating agents [28], have significantly fewer side effects. Although the current treatments for some forms of childhood leukemias have shown significantly improved survival rates, some cases do not respond to standard treatments (chemotherapy, radiotherapy, and stem cell transplantation) or experience relapse of the disease. Many survivors live with various health conditions that are a consequence of the harsh chemotherapy and radiotherapy treatments, highlighting the need for less toxic treatments [27].

Cathepsins, lysosomal cysteine proteases, have emerged as important targets in the development of a variety of therapeutic agents related to induced cell death mechanisms in leukemias [29]. For instance, cathepsins B and L are up-regulated in acute myeloid leukemia (AML) patients in comparison with normal patients, and these conditions show poor overall survival and short disease-free survival [30,31,32]. Patients who achieved complete remission following chemotherapy presented significant reductions in cathepsin L mRNA levels [30]. Furthermore, there are strong associations between high cathepsin L activity in both peripheral blood mononuclear cells (PBMCs), and bone marrow mononuclear cells with poor event-free survival and overall survival rates in pediatric AML [30]. Chronic myeloid leukemia patients in the chronic phase display significantly higher enzymatic activity and mRNA levels of cathepsin L compared to healthy controls and patients suffering from systemic infections [33].

For these reasons, we investigated the effects of APL (miltefosine and three alkylphosphocholine analogs) and alkyltriazoles (compounds **5**–**21**) on human leukemia lineages and observed the cytotoxic effects against Jurkat and CCRC-CEM cells, two ALL lineages. We also characterized the efficacy of these compounds in inhibiting the cysteine protease activity of cathepsins B and L. Molecular docking simulation, molecular dynamics and binding free energy evaluation were performed to determine the interaction of cathepsins and compounds.

## 2. Results

### 2.1. Cytotoxic Potential of Alkyltriazole and Alkylphospholipid Compounds in Different Leukemic Lines

The ability to induce cell death of alkyltriazole and APL compounds was evaluated. The most potent compounds, among the 21 compounds tested, were identified using a high concentration (100 μM) for 24 and 48 h (Figures S1 and S2) in human leukemia lineages. The effect of the compounds was time-dependent, since cytotoxicity was lower at 24 h than at 48 h (Figures S1 and S2). Jurkat and CCRF-CEM cells, ALL cell lineages, were more sensitive to alkyltriazoles and APL compounds than other leukemic lineages; therefore, these lineages were selected for subsequent studies to determine the cell death mechanisms and inhibition of cathepsin proteases.

PBMCs were used as healthy control cells. PBMCS treated with compounds **C9** and **C21** were significantly less sensitive to the effects of the compounds than the tumor lineages. These compounds were less cytotoxic than vinblastine after 24 h of exposure at different concentrations (Figure S3).

### 2.2. Determination of Induction of Cell Death by the Different Compounds

**Figure 2:**
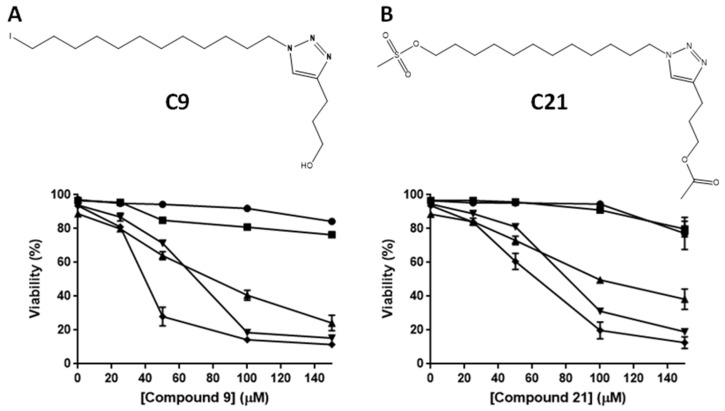
Effects of the alkyltriazole compounds **C9** and **C21** on the viability of different myeloblastic and lymphoblastic leukemia lineages. Lineages cells at 2 × 10^5^ cells/mL were used. Cells were exposed to concentrations ranging from 0 to 150 µM for 24 h, and cell viability was assessed by annexin V-FITC/PI staining and flow cytometric analysis. (**A**) Chemical structure of **C9**, with a hydroxyl group and an iodine atom as substituents. (**B**) Chemical structure of **C21**, with an acetate group and a methanesulfonate as substituents. ⬤ → Kasumi-1 (acute myeloblastic leukemia), ■ → K562 (chronic myeloblastic leukemia), ▲ → ARH-77 (plasma cell leukemia), ▼ → Jurkat (acute lymphoblastic leukemia); ⯁ → CCRF-CEM (acute lymphoblastic leukemia). These results are the mean ± SEM of three independent experiments performed in triplicate.

The EC_50_ values of compounds **C9** and **C21** in the leukemic lineages were determined by concentration–response curves (Figure 2A,B and Table 1). The most sensitive cell lines in the assay were CCRF-CEM, Jurkat and ARH-77 cells with EC_50_ values of 40 μM, 63 μM and 71 μM, respectively, for compound **C9** (Figure 2A, Table 1), and EC_50_ values of 60 μM, 77 μM and 96 μM, respectively, for compound **C21** (Figure 2B, Table 1).

**Figure 3:**
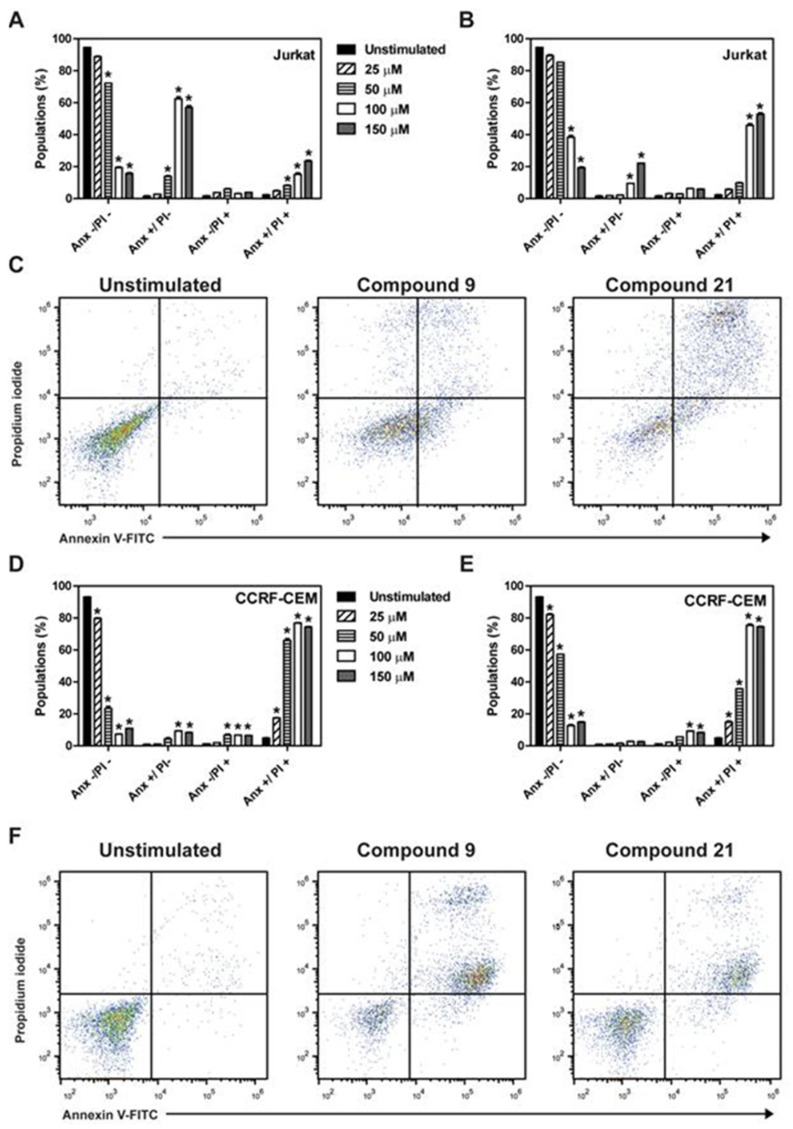
**C9** and **C21** compounds promoted cell death through different mechanisms in Jurkat and CCRF-CEM ALL lineages. Cells of Jurkat and CCRF-CEM at 2 × 10^5^ cells/mL were used. Cells were exposed to concentrations ranging from 0 to 150 µM of **C9** and **C21** for 24 h. Cytotoxicity was evaluated by flow cytometry with annexin V-FITC/PI staining. (**A**,**B**) Quantification of Jurkat cell death treated with **C9** and **C21**, respectively. (**C**) Representative flow cytometry dot plots for untreated controls, **C9** treatment (50 µM) and **C21** treatment (100 µM). (**D**,**E**) Quantification of CCRF-CEM cell death treated with **C9** and **C21**, respectively. (**F**) Representative flow cytometry dot plots for untreated controls, **C9** treatment (50 µM) and **C21** treatment (100 µM). These results are the mean ± SEM of three independent experiments performed in duplicate.

Flow cytometry analysis showed that the treatment of Jurkat cells with compound **C9** resulted in an increase in annexin V^+^/propidium iodide^−^ (Anx^+^/PI^−^) (apoptosis) and annexin V^+^/propidium iodide^+^ (Anx^+^/PI^+^) (late apoptosis/necrotic-like cell death) (Figure 3A). Compound **C21** promoted an increase in the Anx^+^/PI^+^-induced cell death in Jurkat cells (Figure 3B). Figure 3C shows a representative dot plot of the treatments with the compounds **C9** and **C21** at the concentration of EC_50_ in Jurkat cells. The treatment of CCRF-CEM cells with compound **C9** (Figure 3D) and compound **C21** (Figure 3E) increased Anx^+^/PI^+^. Figure 3F shows the representative dot plots of the treatments at the concentration of EC_50_ in CCRF-CEM cells.

### 2.3. Comparative Analysis of Alkyltriazole Compounds with Inhibitors of Cell Death Pathways

**Figure 4:**
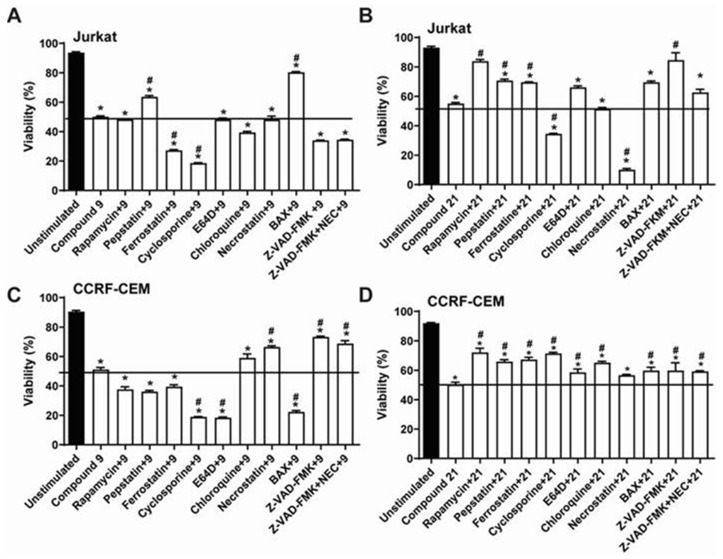
Evaluation of modulators of the pathways involved in the cell death process using specific inhibitors. Jurkat and CCRF-CEM cells at 2 × 10^5^ cells/mL were used. Cells were preincubated with the inhibitors for 1 h. Subsequently, cells were treated for 24 h with the respective EC_50_ of the compounds. Cytotoxicity was evaluated by flow cytometry with annexin V-FITC/PI staining. These results are the mean ± SEM of three independent experiments performed in duplicate. (**A**) Jurkat **C9**, (**B**) Jurkat **C21**, (**C**) CCRF-CEM **C9** and (**D**) CCRF-CEM **C21**. * *p* < 0.05 compared to the negative control. # *p* < 0.05 compared to the compound.

To clarify the mechanism of cell death triggered by compounds **C9** and **C21**, inhibitors of different cellular mechanisms were used. Figure 4 shows the data obtained from the pre-treatment of Jurkat (Figure 4A,B) and CCRF-CEM (Figure 4C,D) cells with different inhibitors of specific cell death pathways, including rapamycin (mTOR), pepstatin-A (acid proteases), ferrostatin-1 (iron-dependent cell death), cyclosporine (calcineurin), E-64d (cysteine protease cathepsins, calpains 1 and 2), chloroquine (lysosomal hydrolases), necrostatin (RIP-1), BAX inhibitor, and Z-VAD-FMK (caspases) in the presence of compounds **C9** and **C21** for 24 h.

The Jurkat cell death promoted by compound **C9** seems to be independent of caspases and non-sensitive to necrostatin, but the inhibitor of BAX, which allosterically prevents BAX channel activation, reduced the cell death elicited by compound **C9**; pepstatin, an inhibitor of aspartyl proteases, partially reduced cell death induced by compound **C9** (Figure 4A).

The results obtained for CCRF-CEM cells were different, as Z-VAD-FMK and necrostatin inhibitors reduced the cell death promoted by compound **C9**; on the other hand, BAX potentiated cell death induced by compound **C9** (Figure 4C).

Cell death promoted by compound **C21** was partially inhibited by rapamycin, pepstatin, and ferrostatin in Jurkat (Figure 4B) and CCRF-CEM lineages (Figure 4D). Z-VAD-FMK and BAX significatively inhibited cell death elicited by compound **C21**, but only in Jurkat cells (Figure 4B). Unexpectedly, necrostatin potentiated cell death triggered by compound **C21** in Jurkat cells (Figure 4B). Cyclosporin, a calcineurin–phosphatase inhibitor, which prevents the mitochondrial permeability transition pore opening, potentiated Jurkat cell death by both compounds, and potentiated cell death by compound **C9** only in CCRF-CEM cells (Figure 4C).

### 2.4. Caspase-3 and PARP Cleavage by Compounds

**Figure 5:**
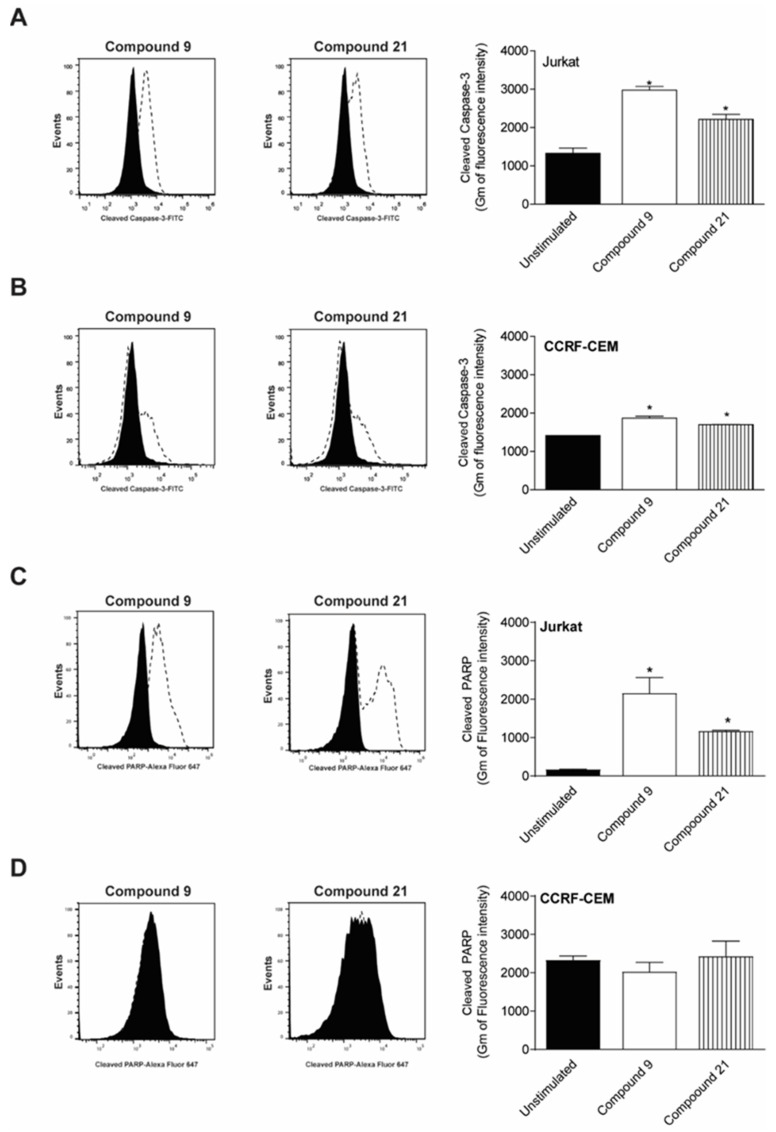
Quantification of cleaved-caspase 3 and -PARP by compounds. Jurkat and CCRF-CEM cells at 2 × 10^5^ cells/mL were used. Subsequently, cells were treated for 24 h with the respective EC_50_ of the compounds. Levels of cleaved caspase-3 conjugated with FITC were quantified by flow cytometry in (**A**) Jurkat and (**B**) CCRF-CEM cells. Representative flow cytometry histograms are showed on the left. Levels of cleaved PARP were assessed using an anti-cleaved PARP conjugated with Alexa Fluor 647 in (**C**) Jurkat and (**D**) CCRF-CEM cells. The geometric mean of fluorescence is shown on the right. The results are mean ± SEM of 3 experiments. * *p* < 0.05 compared to the unstimulated control.

Cleave of caspase-3 and PARP-1, hallmarks of caspase-dependent cell death, were evaluated. Both compounds **C9** and **C21** were able to induce an increase om cleaved caspase-3 in Jurkat cells after 12 h of treatment (Figure 5A), but the presence of cleaved caspase-3 in CCRF-CEM cells was less intense (Figure 5B). Additionally, PARP-1 cleavage induced by compounds **C9** and **C21** was also observed after 12 h of treatment for Jurkat cells (Figure 5C), although activation of PARP-1 in CCRF-CEM cells did not occur (Figure 5D).

### 2.5. Release of Cytochrome c and Nuclear Changes

**Figure 6:**
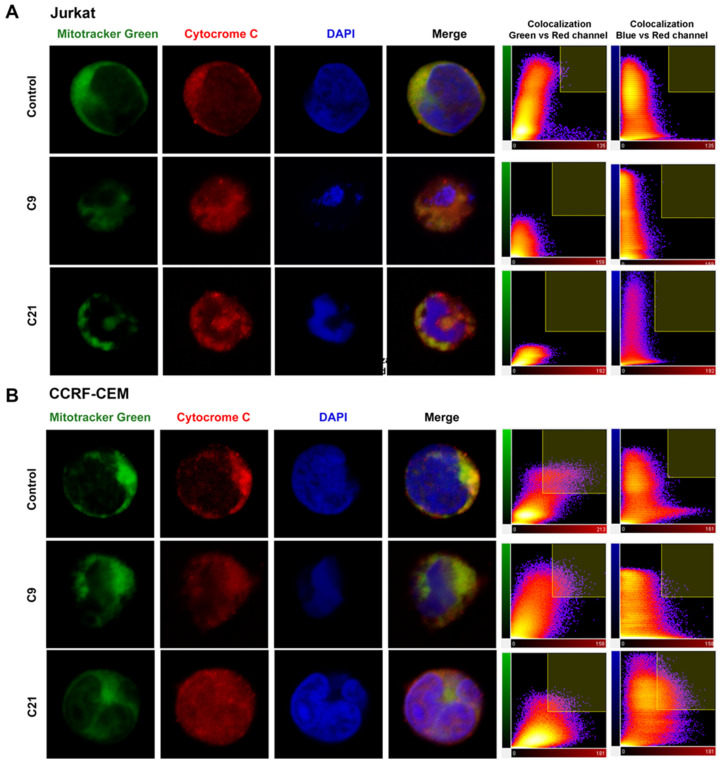
Release of cytochrome C and morphological alterations by compounds. The cells were labeled with MitoTracker Green, anti-cytochrome *c* antibody/Alexa Fluor 594 and DAPI. Cells were imaged using confocal fluorescence microscopy. Representative single-cell images for each condition (untreated control, **C9** and **C21**) are shown. (**A**) Jurkat cells, (**B**) CCRF-CEM cells. Green: Mitotracker green, fluorescent mitochondrial stain; Red: cytochrome C release stain; Blue: DAPI (4′,6-diamidino-2-phenylindole), fluorescent DNA stain; Merge: color overlay images of all stains. The colocalization coefficient among the red, green and blue channels is showed on the right.

Figure 6A (Jurkat cells) and 6B (CCRF-CEM) obtained by confocal microscopy showed the presence of cellular alterations. Color overlay images of all stains (with merged colors) show that Jurkat and CCR-CEM cells treated with compounds **C9** and **C21** lead to the release of cytochrome c (red) throughout the cytoplasm and nucleus region, and not only in mitochondria (green). The colocalization coefficient between the green and red channel was 25–35% in the control cells and after treatment, the colocalization index was reduced from 0 to 5% by compounds **C9** and **C21** in Jurkat cells, but in the CCRF-CEM lineage, the colocalization coefficient remained high (30–40%). On the other hand, the colocalization coefficient between the blue and red channel was 0% in the control cells, and after treatment, the index was increased mainly by compounds in the CCRF-CEM lineage.

Alterations in nuclear morphology were noticed after treatment with the compounds. Compounds **C9** and **C21** caused DNA fragmentation in Jurkat cells, while in CCRF-CEM cells, the compounds caused shrinkage of the nucleus, with the apparent condensation of DNA (Figure 6A,B).

### 2.6. Inhibitory Potential (IC_50_) Evaluation and Mechanism Determination of Compounds with Cathepsins B and L

Since their discovery, lysosomes and lysosomal proteases, including cathepsins such as cathepsin B and L, have often been linked with cell death [34,35]. In this sense, we evaluated the inhibition of cathepsins B and L by the compounds.

The determination of IC_50_ values of alkylphospholipids and alkyltriazoles in the inhibition of cathepsins B and L (Table 2) showed that compounds **C9** and **C21** were the most efficient at inhibiting cathepsin B with IC_50_ values of 3.5 ± 0.2 µM and 3.9 ± 0.2 µM, respectively, and cathepsin L with IC_50_ values of 1.3 ± 0.1 µM and 1.6 ± 0.1 µM, respectively. Interestingly, both compounds were better at reducing the viability of Jurkat and CCRF-CEM cells (Figures S1 and S2).

**Figure 7:**
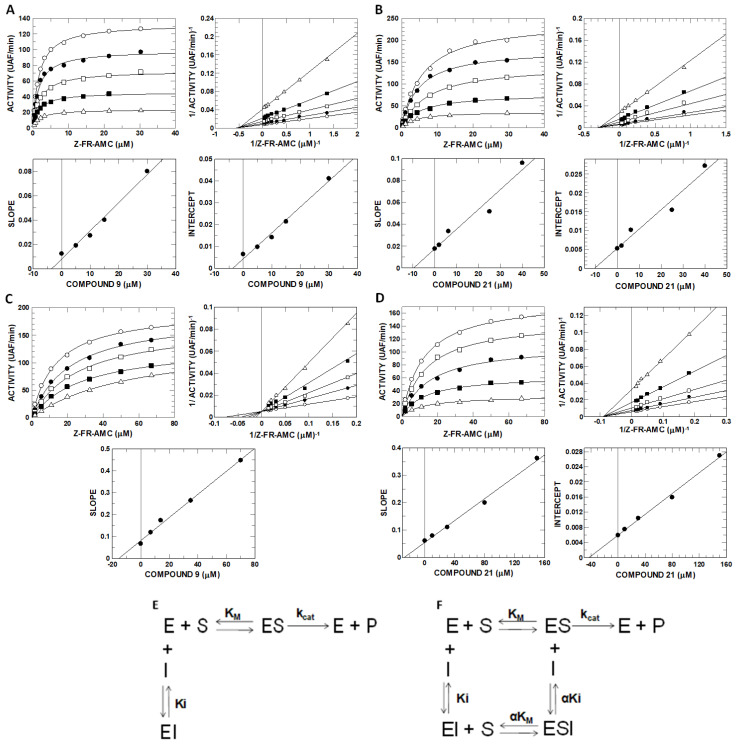
Kinetic inhibition mechanism evaluation and inhibition constant determination of compounds **C9** and **C21** with cathepsins B and L. Compounds **C9** and **C21** were assayed against cathepsins B and L at different concentrations of the substrate Z-FR-AMC and compounds. (**A**) Cathepsin L and compound **C9**; (**B**) Cathepsin L and compound **C21**; (**C**) Cathepsin B and compound **C9**; (**D**) Cathepsin B and compound **C21**; (**E**) Simple linear competitive mechanism that provides only the *K*_i_ profile from (**C**,**F**) Simple linear noncompetitive mechanism that provides the *K_i_* and α*K*_i_ profile from (**A**,**B**,**D**).

**Figure 8:**
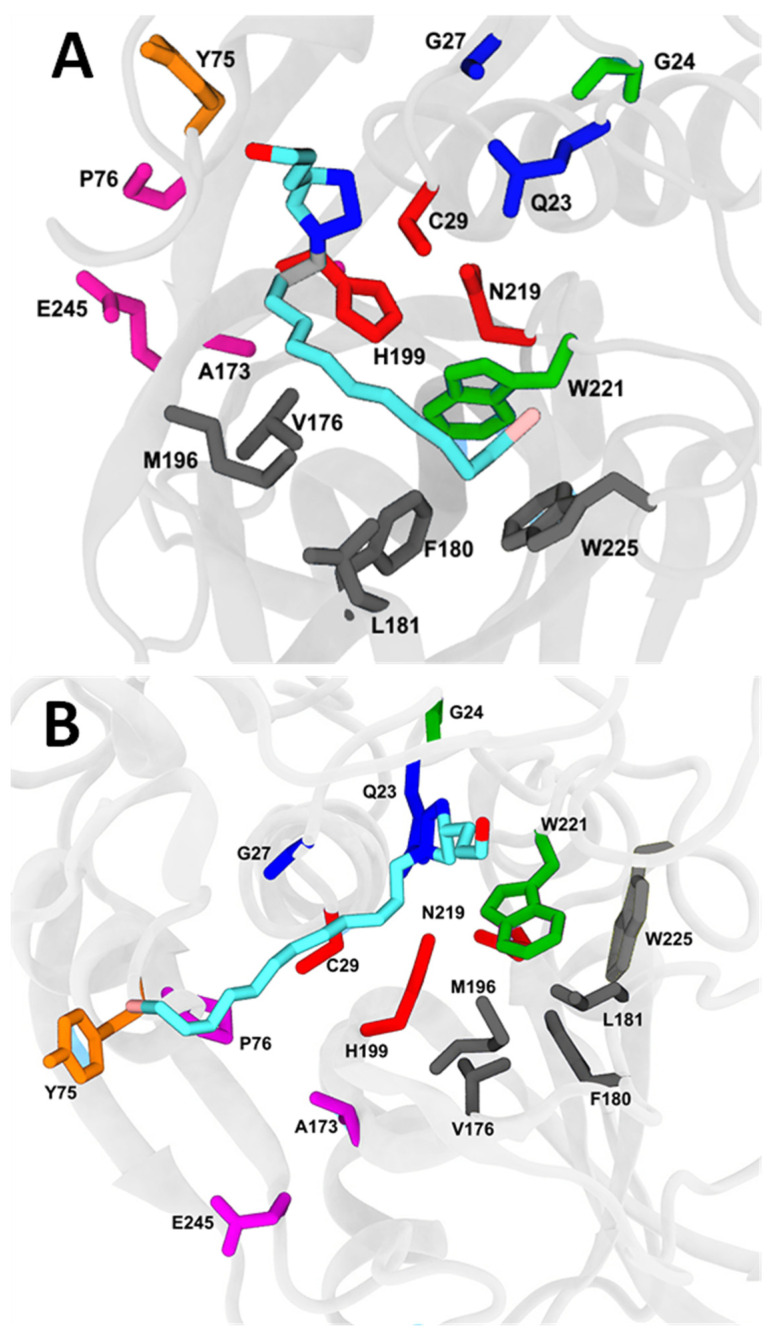
Molecular docking of cathepsin B with compounds **C9** and **C21**. (**A**) Cathepsin B-**C9** complex. (**B**) Cathepsin B-**C21** complex. Catalytic triad in red (Cys29, His199; Asn219). Amino acid subsites: S’3 in green (G24; W221), S’2 in black (M196, L181, V176, F180; W225), S1 in blue (Q23; G27), S2 in magenta (P76, H173; E245) and S3 in orange (Y75).

The compounds **C9** and **C21** were selected for determining the mechanism of inhibition. Accordingly, compound **C9** presented simple linear noncompetitive inhibition mechanisms for cathepsin L (Figure 7A), binding to free enzyme E and complex ES to form EI and EIS (Figure 7F), with *K*_i_ = 3.56 ± 0.26 µM and α*K*_i_ = 3.66 ± 0.07 µM and α ≈ 1, and simple linear competitive inhibition mechanisms for cathepsin B (Figure 7C), binding exclusively to free enzyme E to form EI instead of IES (Figure 7E), with *K*_i_ = 15.3 ± 0.6 µM (Table 3). Compound **C21** presented only a simple linear noncompetitive inhibition mechanism (Figure 7F and Table 3) for both proteases, with *K*_i_ = 9.72 ± 1.23 µM, α*K*_i_ = 10.47 ± 1.17 µM and α ≈ 1 for cathepsin L (Figure 8B) and *K*_i_ = 27.4 ± 1.2 µM, α*K*_i_ = 43.9 ± 1.7 µM and α = 1.6 for cathepsin B (Figure 7D and Table 3).

From the *K*_i_ values, it can be observed that compounds **C9** and **C21** present higher affinity to cathepsin L than cathepsin B, which can be explained by the presence of occluding loops in cathepsin B. The occluding loop appears to play an important role in the binding of substrates that are cleaved by cathepsin B [36] and it is for this reason that it would be expected to interfere with the binding of inhibitors.

### 2.7. Molecular Docking and Molecular Dynamics

Since the compounds can inhibit both cathepsins, we performed molecular docking and molecular dynamics evaluations. Protein interactions with the ligand can be monitored throughout the simulation. These interactions can be categorized by type and summarized, as shown in Figure S4. Protein–ligand interactions were categorized into the following four types: hydrogen bonds and hydrophobic, ionic and water bridges.

During the analysis of the bonding pattern between the residues and ligands, we observed a significant role of hydrophobic interactions, and water bridges along with hydrogen bonds.

**Figure 9:**
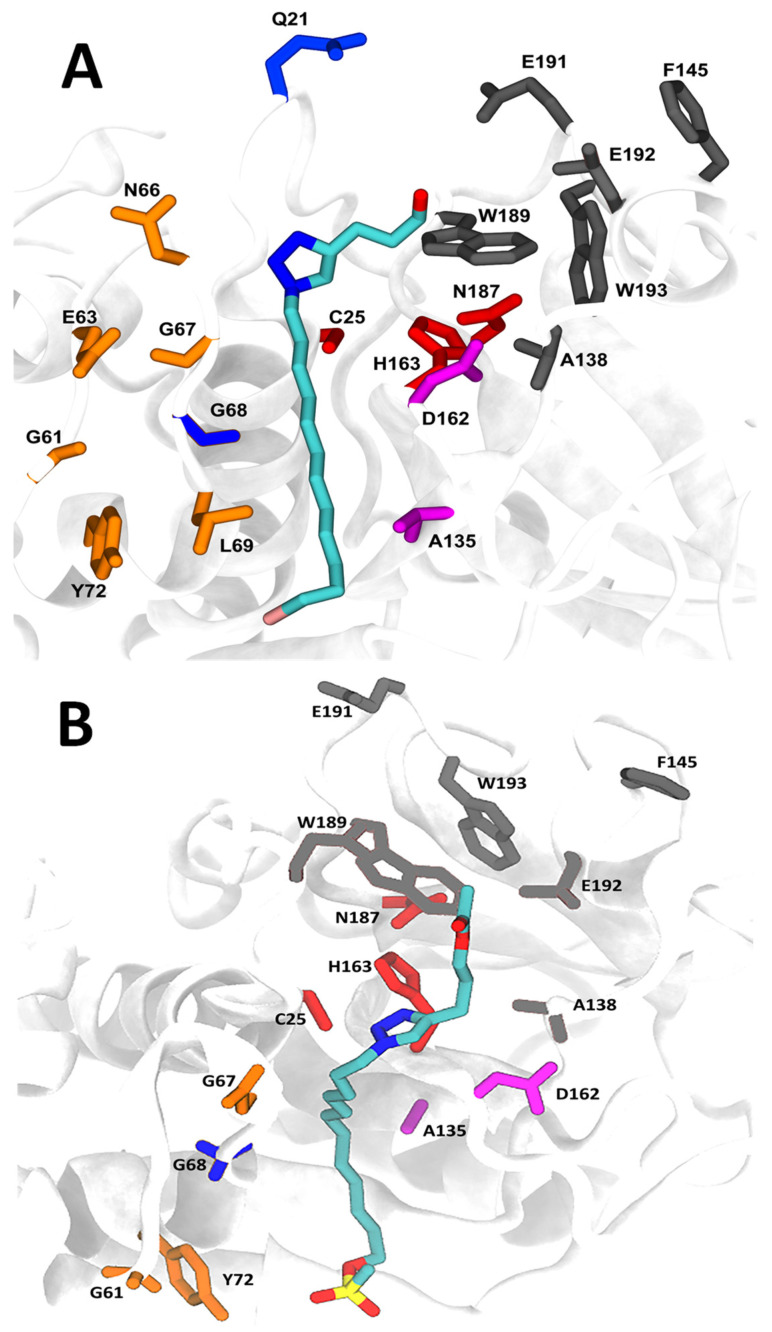
Molecular docking of cathepsin L with compounds **C9** and **C21**. (**A**) Cathepsin L-**C9** complex. (**B**) Cathepsin L-**C21** complex. Catalytic triad in red (Cys25, His163; Asn187). Amino acid subsites: S’3 in deep purple (A135; D162), S’2 in black (W189, A138, E192, W193, F145; E191), S1 in blue (G68, Q21), S2 in magenta; S3 (N66, E63, G67, G61, L69; Y72) in orange.

Molecular docking of compounds **C9** and **C21** and cathepsins B and L is presented in Figure 8 and Figure 9, respectively, in addition to the main amino acids of the interactions. The binding free energies determined by generalized Born and surface area solvation (MM-GBSA) were −27.05 ± 1.21 kcal·mol^−1^ and −18.27 ± 0.98 kcal·mol^−1^ for cathepsin B-**C9** (Figure 8A) and cathepsin B-**C21** (Figure 8B) complexes, respectively, and −34.68 ± 1.33 kcal·mol^−1^ and −26.37 ± 2.01 kcal·mol^−1^ for cathepsin L-**C9** (Figure 9A) and cathepsin L-**C21** (Figure 9B) complexes, respectively. These data are in accordance with IC_50_ (Table 2) and *K*_i_ values (Table 3), where the complex cathepsin L-**C9** presented the best inhibitory potential (IC_50_) and inhibitory constant of bind affinity (*K*_i_). We also determined binding energies based on the inhibition constant *K*_i_ of compounds **C9** and **C21** for cathepsins. Values of −46.37 kcal·mol^−1^, −42.66 kcal·mol^−1^, −40.98 kcal·mol^−1^ and −38.82 kcal·mol^−1^ were obtained for the complexes cathepsin L-**C9**, cathepsin L-**C21**, cathepsin B-**C9** and cathepsin B-**C21**, respectively, thus corroborating the binding energy data from the MM-GBSA analysis.

Cathepsin B-**C9** presented 11 significant water bridges with Gly27, Ser28, Cys29, Cys67, Gly68, Asp69, Gly70, Gly198, His199, Trp221 and Asn222, and 9 types of hydrophobic interactions with amino acids Cys29, Trp30, Tyr75, Val176, Phe180, Leu181, Met196, Trp221 and Trp225 (Figure S4A). However, the cathepsin B-**C21** complex showed eight examples of water binding with Gly24, Cys26, Cys29, Cys108, His110, His111, Trp221 and Asn222 and five hydrophobic bonds with Val112, Leu181, Met196, Trp221 and Trp225 (Figure S4B).

Accordingly, compound **C9** displayed six types of exclusively hydrophobic interactions with cathepsin L at amino acids Trp26, Leu69, Ala135, Leu144, Phe145, Trp193 (Figure S4C).

In the cathepsin L-**C9** complex (Figure S4D), hydrophilic interactions were predominant regarding the binding affinity, where 15 water bridges with Gln21, Cys25, Gly61, Glu63, Cys65, Asn66, Asp71, Tyr72, Asp114, Asp137, Glu141, Glu159, Asp160, Met161 and Asp162, and 10 types of significant hydrophobic interactions with amino acids Trp26, Leu69, Tyr72, Ala135, Leu144, Phe145, Met161, Trp189, Trp193 and Ala214 were observed. On the other hand, the cathepsin L-**C21** complex promoted the formation of 10 water bridges with amino acids Gln21, Cys25, Ala138, Glu141, Leu144, Phe145, Lys147, Asp162, Trp189 and Glu192, and only 4 types of hydrophobic interactions with Leu144, Phe145, Trp189 and Trp193. Top poses were selected for compounds **C9** and **C21** and cathepsins B and L (Figure S5), and averages of RMSD (Figure S6A) and the radius of gyration (Figure S6B) of compounds and cathepsins were determined. The complexes cathepsin L/**C9**, cathepsin L/**C21**, cathepsin B/**C9** and cathepsin B/**C21** were stable after 100ns. The average center of mass distances of **C9** was 4.2 Å for cathepsin B and 3.78 Å for cathepsin L, while the **C21** center of mass distances was 4.0 Å for cathepsin B and 3.9 Å for cathepsin L (Figure S6).

By analyzing the data of catalytic cysteine of both cathepsins, it was evident that cysteine-29 showed 59.7% and 81.25% of the ionic bonds and 4.7% and 8.6% of the H-bonds at the interaction fraction in the complexes cathepsin B-**C9** and cathepsin B-**C21**, respectively. The catalytic cysteine-25 showed 45.7% and 42.1% of the H-bonds at the interaction fraction in the complexes cathepsin L-**C9** and cathepsin L-**C21**, respectively.

## 3. Discussion

Although the use of chemotherapy has been reported since the mid1900s, high mortality rates are still related to treatment failure, since anti-cancer therapy is currently still not fully effective or safe [37]. Current therapeutic strategies include a variety of chemotherapeutic drugs and hormonal agents that interfere with cell homeostasis and induce certain cell types to die, most commonly highly mitotic cells [38].

Unlike the classical chemotherapeutic agents that exert their therapeutic effects by promoting DNA damage, the APL group, which includes miltefosine, edelfosine and perifosine, is a group of structurally similar APLs that act on the membrane of tumor cells [39]. Phospholipids such as edelfosine contain the chemical ether group in their structure, which forms stable bonds following cell membrane blocking membrane turnover, interrupting lipid-dependent signaling pathways and inducing cell death [15].

With this perspective, the present study investigated the biological activity of alkylphospholipid and alkyltriazole derivatives in models of hematological malignancies and analyzed the mechanism involved in the induction of cell death and inhibition of cathepsins B and L. Initially, screening was performed to verify the cytotoxic effect of the 21 compounds in the hematological neoplasia lineages (Kasumi-1, K562, ARH-77, CCRF-CEM and Jurkat). The compounds were more selective and more effective for the ALL lineages (Jurkat and CCRF-CEM). The alkylphospholipid- and alkyltriazole-derivative compounds that presented the highest cytotoxicity in the ALL lineages (Jurkat and CCRF-CEM) were **C9** and **C21**. The antitumor effect of erufosine, an alkyltriazole derivative, was demonstrated and compared with miltefosine and turmeric in T-cell lymphomas lineages with an EC_50_ of approximately 60 μM, suggesting that erufosine is more effective than miltefosine [40]. Our data are in accordance with these previous findings, as compound **C9** presented EC_50_ values from 40 to 70 μM in Jurkat and CCRF-CEM lineages. The EC_50_ values found for miltefosine against the studied lines varied between 120 and 200 μM. The highest EC_50_ value observed for miltefosine was still below the maximum concentration allowed for local therapy, since miltefosine is approved for local treatment of cutaneous metastasis of breast cancer as a 6% solution [41].

The antineoplastic mechanisms of action of alkylphosphocholines include the inhibition of phosphatidylcholine biosynthesis [1,2,39] and the induction of apoptosis by inhibition of the PI3K/Akt/PKB pathway [42], leading to reduced cell survival or increased apoptosis, mediated either through inducing intracellular stress (reactive oxygen species), by blocking essential survival signals, or by inducing various pro-apoptotic cell signaling pathways [39].

Alkylphospholipids represent a class of drugs that do not interact directly with DNA, but instead accumulate on the cell membrane, interfering with lipid metabolism and signaling pathways and leading to apoptosis and autophagy [15]. Treatment of CEM-R cells, an acute T-cell lymphoblastic leukemia, with alkylphospholipid perifosine activate multiple caspases, including caspases-3,8,9, and promote the cleavage of PARP, producing apoptosis [43]. The mechanism triggered by compounds **C9** and **C21** that causes cell death in Jurkat and CCRF-CEM cells is not similar and showed differences between lineages. Compound **C9** induced an increase in Anx^+^/PI^−^ and Anx^+^/PI^+^ cells, cleaved caspase 3 and cleaved PARP and the release of cytochrome C with DNA fragmentation in Jurkat cells. Moreover, the cytotoxicity of compound **C9** was sensitive to pepstatin and Bax. Similarly, induced cell death by **C21** affected Anx^+^/PI^−^ and Anx^+^/PI^+^ cells, cytochrome C release, cleaved caspase 3 and cleaved PARP and DNA fragmentation in Jurkat cells, but induced cell death was blocked by more inhibitors, such as rapamycin, pepstatin, ferrostatin, E64, Bax and Z-VAD-FMK. These results suggested that **C9** and **C21** produce preferentially activation-induced cell death with the characteristics of apoptosis in Jurkat cells.

The CCRF-CEM lineage favored Anx^+^/PI^+^ cells and demonstrated a small increase in cleaved caspase 3 and PARP, without the release of cytochrome C, but with nuclear morphological alterations, such as necrotic induced cell death. However, necrostatin and Z-VAD-FMK partially inhibited induced cell death by compound **C9** in the CCRF-CEM lineage. In contrast, cell death induced by compound **C21** was inhibited. Thus, the effect of compounds **C9** and **C21** produced activation-induced cell death by apoptosis and necrotic-like cell death for the ALL lineage.

However, given the demand of new drugs against cancer, there is a need for new drugs with preferential apoptotic potential against tumor cells. Our study indicates that there is an additional complicating factor when identifying novel compounds with therapeutic potential. The data indicate that the effects of the compounds vary among the different cell types, indicating that molecules interact in different ways depending on the different cell types, thereby potentially producing distinct effects. For instance, the alkaloid 4-Deoxyraputindole C, extracted from the plant *Raputia praetermissa*, caused cell death due to its apoptotic characteristics in Raji cells, a human lymphoma lineage, and cell arrest in A549 cells, a human lung carcinoma lineage [44]. Antimicrobial peptides such as gomesin produce apoptosis in K562 cells, a human chronic myeloid leukemia, and necrosis-like cell death in B16 cells, a murine melanoma lineage [45,46]. Polymatin B is a sesquiterpene lactone, isolated from the leaves of *Smallanthus sonchifolius*, that induces different cell death mechanisms in the three tumor cells tested [47]. Concentrations of the compound also are important to its activity, for instance, Canthin-6-one, a beta-carboline alkaloid, causes cell death, cell arrest and cell differentiation depending on the concentration used [48,49].

Despite the differences in the mechanism observed for compounds **C9** and **C21**, the cytotoxic activity of these compounds is important, particularly in ALL lineages. T-cell ALL is an aggressive form of leukemia that accounts for about 25% and 15% of ALLs in adult and pediatric cohorts, respectively [50,51,52]. T cell-ALL arises from T-cell progenitors in the thymus, expressing immature T-cell immunophenotypic markers [50]. Patients with T-ALL usually have high white blood cell counts and may present organomegaly [53]. The biology and pathogenesis of T-ALL are not yet fully understood [50], since each T-ALL case contains possibly more than 10 biologically relevant genomic lesions, each contributing to the transformation of normal T-cells into an aggressive leukemia cell with impaired differentiation, improved survival and proliferation characteristics, and altered metabolism, cell cycle and homing properties [54]. The prognosis of this disease has improved with the intensified chemotherapy available [52]. Cure rates in modern protocols reach about 50% in adults and 75% in children with T-ALL. However, the outcome of patients with primary resistant or relapsed leukemia is poor, mainly for T-ALL adult patients [51,52]. Therefore, the development of novel and effective treatment strategies for this disease is necessary [51,52]. In this way, the compounds **C9** and **C21** can be a future alternative treatment for this kind of disease.

Several studies have suggested the potential impact of proteases in AML patients, and it is known that cathepsin B and cathepsin L are able to convert pro-urokinase plasminogen activators to their active forms, which are also highly expressed in AML and are associated with a poor prognosis [55]. The increased enzymatic activity of cathepsin L and cathepsin B in pediatric AML patients and the high activity of these cathepsins are negatively correlated with event-free survival and overall survival of the patients [56]. Pandey et al. [31] demonstrated a significant increase in cathepsin B and L activity in AML patients when compared to healthy controls [30,31]. Moreover, inhibition of cathepsin B activity induced apoptosis in AML cells, suggesting the contribution of this protease in the pathogenesis of this malignancy, but participation of cathepsins in other leukemias, such as ALL, deserve further investigation.

Deletion of *cathepsins B and/or L* reduces tumor vascularization, significantly increases tumor cell death, decreases tumor cell proliferation, impairs tumor invasion, promotes preservation of E-cadherin protein, establishes cell–cell junctions and reduces pro invasive functions of cathepsins [57]. In this way, compounds **C9** and **C21** presented the best IC_50_ values in the inhibition of cathepsins B and L, interacting well with both enzymes. In addition, compounds **C9** and **C21** had greater affinity for cathepsin L, *K*_i_ = 3.56 ± 0.26 µM and *K*_i_ = 9.72 ± 1.23 µM, respectively, than cathepsin B, *K*_i_ = 15.3 ± 0.6 µM and *K*_i_ = 27.4 ± 1.2 µM, respectively. Enzymatic increased cathepsin L and cathepsin B activity and respective mRNA levels were demonstrated in pediatric patients with AML [56]. High activity of both proteases negatively correlated with event-free survival and overall survival in these patients.

## 4. Materials and Methods

### 4.1. Alkylphospholipid and Alkyltriazole Compounds

Alkylphospholipid and alkyltriazole compounds (Table S1) were synthetized as previously described [18]. The compounds of this study were evaluated for their leishmanicidal and antitumor activity (colon carcinoma and uterine carcinoma) [17,18]. Stock solutions of all compounds were prepared in DMSO, stored at −20 °C and diluted in culture medium before use. The final concentration of DMSO in the culture medium was never higher than 0.25%. DMSO was used as a vehicle control at the same concentration as that used with the compounds. The compounds were diluted in the cathepsin buffer for the enzymatic assays.

### 4.2. Cell Cultures

Human leukemia cell lines (Kasumi-1, K-562, ARH-77, Jurkat and CCRF-CEM, see Table S2) were purchased from American Type Culture Collection-ATCC (Manassas, VA, USA). The cells were maintained in RPMI 1640 (Sigma-Aldrich, Burlington, MA, USA) culture medium, supplemented with 10% fetal bovine serum, 100 U/mL penicillin (Sigma-Aldrich, Burlington, MA, USA) and 100 µg/mL streptomycin (Sigma-Aldrich, Burlington, MA, USA). Cells were cultured in a *humidified* atmosphere at 37 °C and 5% CO_2_.

### 4.3. Peripheral Blood Mononuclear Cell Culture

Blood was collected using EDTA Vacuum Blood Collection Tubes. Peripheral blood mononuclear cells were isolated from three normal healthy individuals after they provided informed consent and after prior approval by the local Ethics Committee of University of Mogi das Cruzes (ethical appreciation number 72986617.3.0000.5497, and approval number 2.236.397). The fraction of mononuclear cells was separated from the blood by centrifugation with Ficoll-Histopaque-1077 (density 1.077 g/mL, Sigma-Aldrich, Burlington, MA, USA) at 400× *g* for 30 min. Mononuclear cells were maintained in 100 U/mL penicillin and 100 mg/mL streptomycin in an incubator with a humidified atmosphere at 37 °C and 5% CO_2_.

### 4.4. Cellular Viability Using Calcein-AM and EthD-1 (Live/Dead Assay)

For initial screening, the cells (2 × 10^5^ cells/mL) were treated with 100 μM of each compound for 24 and 48 h. For measurement of cellular viability, 1 μmol/L calcein-AM, a cell permeant green fluorescent indicator, and 4 μmol/L EthD-1, a non-permeant red fluorescent indicator, were added and then incubated for 20 min at 5% CO_2_ and 37 °C. Sample analysis was performed using an Accuri C6 flow cytometer (BD Biosciences, San Jose, CA, USA) with an acquisition value of 10,000 events.

### 4.5. Annexin V/propidium Iodide Assay

Leukemia or PBMC cells (2 × 10^5^ cells/mL) were incubated for 24 h in both the absence and presence of compounds. PBMCs were also treated with vinblastine (Sigma-Aldrich, Burlington, MA, USA) for 24 h as the positive control. Then, the cells were centrifuged and resuspended in binding buffer (0.01 M HEPES, pH 7.4, containing 0.14 M NaCl, and 2.5 mM CaCl_2_) and incubated at room temperature with annexin V-FITC (BD Biosciences, San Jose, CA, USA) and 5 µg/mL PI (Sigma-Aldrich, Burlington, MA, USA) for 20 min. Sample analysis was performed using an Accuri C6 flow cytometer (BD Biosciences, San Jose, CA, USA) with the acquisition value of 10,000 events.

To investigate the mechanisms of compound-induced cell death, cells were pretreated with the following inhibitors for 1 h: cyclosporine (20 μM), BAX channel blocker (20 μM), rapamycin (20 μM), ferrostatin (20 μM), chloroquine (20 μM), pepstatin A (20 μM), E64 (20 μM), Z-VAD-FMK (20 μM) and necrostatin-1 (20 μM). All inhibitors were purchased from Tocris Bioscience (Abingdon, OX, UK).

### 4.6. Caspase- 3 and Poly(ADP-ribose) Polymerase-1(PARP) Cleavage

Jurkat and CCRF-CEM cells (2 × 10^5^ cells/mL) were treated with **C9** and **C21** compounds for 12 h. Then, the cells were centrifuged, washed, fixed with 2% paraformaldehyde in PBS for 30 min and permeabilized with 0.01% saponin in PBS for 15 min at room temperature. Afterwards, the cells were incubated for 1 h at 37 °C with anti-cleaved-caspase 3-FITC monoclonal antibodies (Cell Signaling, Danvers, MA, USA) or mouse anti-cleaved PARP-PE/CF594 (Asp^214^) antibodies (BD Biosciences, San Jose CA, USA). After incubation, the fluorescence was analyzed using an Accuri C6 flow cytometer (BD Biosciences, San Jose, CA, USA). A total of 10,000 events were acquired per sample.

### 4.7. Cytochrome C Release and Nuclear Alteration Analyses

The release of cytochrome *c* from the mitochondria was estimated by using double-labeled confocal cell imaging with MitoTracker Green (Invitrogen, Waltham, MA, USA) and a cytochrome *c* antibody. Jurkat and CCRF-CEM cells (2 × 10^5^ cells/mL) were incubated with **C9** and **C21** compounds for 12 h. Then, cells were incubated with 100 nM MitoTracker Green (Invitrogen, Waltham, MA, USA) for 30 min at 37 °C. Following this, cells were washed with PBS, fixed with 2% paraformaldehyde in PBS for 30 min and permeabilized with 0.01% saponin in PBS for 15 min. Then, the cells were incubated with a monoclonal rabbit antibody against human cytochrome *c* at 1:100 dilutions (0.5 μg/mL, Cell Signaling, Danvers, MA, USA) for 2 h at room temperature. After washing, cells were incubated for 40 min with anti-rabbit IgG conjugated with Alexa Fluor 647 at 1:500 dilutions (Invitrogen, USA) and 1.0 μg/mL DAPI (Sigma-Aldrich, Burlington, MA, USA). Microscopy analyses were performed using a confocal laser scanning microscope (Leica SP8 Confocal Microscope, Wetzlar, HE, Germany) with an x63 objective under oil immersion. Alexa Fluor 647 was excited using a HeNe laser (λ_Ex_ = 633 nm) and its emissions were detected at 650–700 nm. MitoTracker Green was excited using an argon laser (λ_Ex_ = 488 nm) and its emissions were detected at 500–550 nm. DAPI was excited with a diode laser (λ_Ex_ = 405 nm) and its emissions were detected at 370–460 nm. Additionally, DAPI staining was used to observe the apoptotic nuclear morphology.

The quantitative evaluation of colocalization was performed by using the percentage of colocalized between channels, which correspond to the pixels with red fluorescence and green or blue fluorescence. A threshold of 70 (on a scale of 8 bits by channel) was used. The colocalization coefficient was calculated using the Imaris software.

### 4.8. Determination of IC_50_ Values for Inhibitors

Cathepsin L was assayed in a reaction buffer of 100 mM sodium acetate, containing 100 mM NaCl, 5 mM EDTA and 20% glycerol at pH 5.5. Cathepsin B was assayed in the same buffer supplemented with 0.01% Triton X-100. The enzymes were pre-incubated in the presence of 3 mM DTT for 5 min at 37 °C in a 1 mL final volume with constant stirring. The enzyme activities were monitored using the fluorogenic probe Z-FR-AMC (50 µM and 5 µM for cathepsin B and L, respectively), and the fluorescence was monitored by spectrofluorometry using the fluorometer RF6000 (Shimadzu, Tokyo, Japan) set to λ_Ex_ = 360 nm and λ_Em_ = 480 nm. The inhibitory potential values (*IC*_50_) were progressively increased with the concentration of the compounds and *IC*_50_ values were calculated by nonlinear regression. The data were analyzed by Grafit 5.0.13 software [58] using Equation (1).

(1) $$y=\frac{100\%}{1+{\left(\frac{x}{IC_{50}}\right)}^{S}}$$

### 4.9. Enzyme Kinetics and Determination of the Mechanism of Inhibition

Studies of cathepsins B and L inhibition kinetics were performed with different concentrations of Z-FR-AMC, ranging from 0 to 5.6 and 0 to 46.3 µM for cathepsin L and cathepsin B, respectively, in the presence and absence of compounds. For every kinetic measurement, the compounds were pre-incubated with each enzyme for 10 min before adding the substrate. All kinetic assays were carried out in duplicate. Inhibition constants were determined using different equations, depending on the inhibition mechanism [59]. The assumed *K*_M_ values of cathepsin B and cathepsin L for Z-FR-AMC were 23.4 µM and 2 µM, respectively, as previously described [44]. The data of the activity rate and substrate concentration generated rectangular hyperbolic profiles that were linearized using the Lineweaver–Burk/double reciprocal plot. The replot profiles of the slope vs inhibitor and intercept vs inhibitor provided the *K_i_* and α*K_i_* parameters, respectively.

### 4.10. Molecular Docking Simulation

To find the binding site of the target specific region of the best compounds, we used molecular docking. Atomic coordinates of cathepsin B complexed with dipeptidyl nitrile inhibitor (Protein Data Bank:pdb ID 1gmy) and cathepsin L with a nitrile inhibitor (pdb ID 2xu3) were taken from the Protein Data Bank. The structure was preprocessed manually and MGL tools [60] were used for removing co-crystallized ligands from their coordinates and to perform energy minimization, followed by the addition of missing hydrogen atoms to polar groups. Each compound was used for molecular docking analysis against the processed structures of either cathepsin B or L in order to predict the bound conformations and the affinities. As the correct topology of each substrate is unknown, two approaches were taken. The cavity detection-guided blind docking approach [61] was used to sample possible binding sites and the Edock docking tool [62], using replica exchange Monte Carlo simulations, was used to achieve high-quality blind docking. The top-ranking poses were reloaded on the MGL tools and docked using AutoDock Vina [63]. PyMOL [64] and VMD (visual molecular dynamics) [65] were used for the purpose of visualizing the different interactions between the cathepsins and each compound. The AutoGrid settings with 76 × 64 × 68 grid size and a grid spacing of 0.462 Å were used for preparing the grid. All dockings were performed using a Lamarkian genetic algorithm (LGA), using 500 individual LGA runs. Top-ranked poses were selected by symmetry-corrected heavy-atom root mean square deviation (RMSD) calculations. The topology of the top 3 complexes with different binding sites was used for further molecular dynamics analysis. The top pose was selected and subjected to molecular dynamics simulations.

### 4.11. Molecular Dynamics

AmberTools16 and Amber16 were utilized to generate topologies for use in MD. The protein–ligand complexes were solvated in a truncated octahedron box with water at least 10 Å from the complexes. Each system was neutralized by adding Na^+^/Cl^−^ ions at a final concentration of 0.15 M. Atom types and parameters for different compounds were assigned by the general amber force (GAFF) [66] and partial charges for ligands were derived using the AM1-BCC method [67]. The 14SB force field [68] was adopted for both cathepsins, with water molecules being treated with the TIP3P water model [69].

The PMEMD.cuda modules in the AMBER16 package were used to run the simulations [70,71,72]. A set of steps of energy minimization was then performed initially to remove possible steric crashes, with harmonic restraint force constants that were decreased from 20 to 10, 5, 1 and 0 kcal/mol/Å^2^, progressively. Each system was then gradually heated from 0 to the final temperature of 300 K. A 1 fs time step was used for the initial dynamics and heating stages, while a 2 fs time step was set for the remaining equilibrium and production stages. A periodic boundary condition was used to produce a constant temperature and pressure (NPT) ensemble. The pressure was controlled at 1 atm with a pressure relaxation time of 1 ps. The temperature was regulated using Langevin dynamics [73,74], with collision frequency of 5 ps^−1^. The particle mesh Ewald (PME) method [75,76] was employed to handle the long-range electrostatics and a 10 Å cutoff was set to treat real-space interactions. All of the covalent bonds that involved hydrogen atoms were constrained with the SHAKE algorithm [77]. The simulation time for each system was 500 ns in triplicate.

### 4.12. Free Energy Evaluation and Decomposition into Per-Residue Contributions

In total, 1500 ns of MD simulations on the reduced system were collected. The free energy of binding of both compounds to both cathepsins was evaluated by means of the molecular mechanics–generalized Born surface area (MM-GBSA) post-processing method [78]. In these methods, the binding free energy of each compound was evaluated as

(2) $$\Delta G_{bind}=G_{complex}\;-\;(G_{receptor}\;+\;G_{ligand}),$$

*G_complex_ G_receptor_*, *G_ligand_* are the absolute free energies of the complex, receptor, and ligand, respectively, averaged over the equilibrium over a single trajectory of the complex. According to these schemes, the free-energy difference can be expressed as

(3) $$\Delta G_{bind}=\Delta E_{MM}+\Delta G_{solvation}-T{\Delta S}_{conformation},$$

Δ*E_MM_* is the difference in the molecular mechanics energy, Δ*G_solvation_* is the solvation-free energy, and Δ*S_conformation_* is the solute conformational entropy. The first two terms were calculated with the following equations:

(4) $$\Delta E_{MM}=\Delta E_{bond}+\Delta E_{angle}+\Delta E_{torsion}+\Delta E_{vdw}+\Delta E_{elec}$$

(5) $$\Delta G_{solvation}=\Delta G_{GB}+\Delta G_{Surface}$$

*E_MM_* includes the molecular mechanics energy contributed by the bonded (Ebond, Eangle, and Etorsion) and nonbonded (Evdw and Eele, calculated with no cutoff) terms of the force field, and Δ*G_solvation_* is the solvation-free energy, as modeled by the sum of electrostatic contribution, Δ*G_solvation_*, which includes the non-polar energy as evaluated using the MMGBSA and nonpolar contribution, Δ*G_Surface_*, based on the solvent-accessible surface area. Solvation-free energies were calculated using one over three conformations, corresponding to 40,000 frames from the MD simulations.

### 4.13. Statistical Analyses

All the data represent at least three independent experiments and are expressed as mean ± standard error (SEM). Statistical analyses were performed using Student’s *t*-test for comparisons between two groups, and analysis of variance (ANOVA) with Dunnett’s post hoc test for multiple comparisons among the groups. A probability value of *p* < 0.05 was considered significant. GraphPad Prism 5.01 software was used for data analyses.

## 5. Conclusions

In conclusion, alkyltriazole with a simple long chain (**C9** and **C21**) presented cytotoxic activity against leukemia cells and potent effects as an inhibitor of cysteine protease cathepsins, suggesting that it is a promising scaffold for the development of molecules for anti-leukemia applications. These structures deserve more studies to increase their potency against ALL lineages and new investigations into other tumor cells.

## Figures and Tables

**Table 1:** Effects of the alkyltriazole compounds **9** and **21** on different myeloblastic and lymphoblastic leukemia cell lines.

Cell	Compound (EC_50_) µM		
	Miltefosine	9	21
Kasumi	ND	>140	>140
K562	ND	>140	>140
ARH-77	ND	71 ± 0.8	96 ± 1
Jurkat	186 ± 12	63 ± 1	77 ± 1.5
CCRF-CEM	120 ± 3	40 ± 1.2	60 ± 2

ND = not determined.

**Table 2:** Determination of IC_50_ values of alkylphospholipids and alkyltriazoles in the inhibition of cathepsins B and L.

Compound	IC_50_ (µM)	
	Cat B	Cat L
**1**	7.5 ± 0.1	10.5 ± 0.2
**2**	23.7 ± 0.9	30.3 ± 3.3
**3**	20.6 ± 0.4	18.6 ± 0.3
**4**	11.3 ± 0.7	8.8 ± 0.3
**5**	9.9 ± 0.4	18.7 ± 0.5
**6**	23.4 ± 1.1	9.7 ± 0.4
**7**	10.8 ± 0.2	5.3 ± 0.1
**8**	10.9 ± 0.9	8.7 ± 0.3
**9**	3.5 ± 0.2	1.3 ± 0.1
**10**	61.9 ± 4.1	7.5 ± 0.2
**11**	12.3 ± 0.4	1.5 ± 0.1
**12**	4.6 ± 0.3	3.4 ± 0.1
**13**	6.2 ± 0.2	2.2 ± 0.1
**14**	10.5 ± 0.2	2.6 ± 0.1
**15**	11.4 ± 0.5	5.6 ± 0.2
**16**	8.8 ± 0.6	13.5 ± 0.2
**17**	22.7 ± 0.4	7.9 ± 0.2
**18**	13.2 ± 0.3	11.0 ± 0.2
**19**	8.1 ± 0.5	2.5 ± 0.1
**20**	4.4 ± 0.3	4.8 ± 0.1
**21**	3.9 ± 0.2	1.6 ± 0.1

Compound **1**: Miltefosine.

**Table 3:** Kinetic parameters of inhibition of cathepsins B and L by compounds **C9** and **C21**.

Enz.	Compound C9				Compound C21			
	*K*_*i*_ (µM)	α*K*_*i*_ (µM)	α	Mec.	*K*_*i*_ (µM)	α*K*_*i*_ (µM)	α	Mec.
Cat L	3.56 ± 0.26	3.66 ± 0.07	1	SLNC	9.72 ± 1.23	10.47 ± 1.17	1	SLNC
Cat B	15.3 ± 0.6	--	--	SLC	27.4 ± 1.2	43.9 ± 1.7	1.6	SLNC

Enz = enzyme; Mec = mechanism; SLNC = simple linear non-competitive; SLC = simple linear competitive. α factor reflects the change promoted by the binding of the inhibitor to subsequent substrate binding (and vice versa).

## Data Availability

Not applicable.

## Supplementary Materials

The following supporting information can be downloaded at: https://www.mdpi.com/article/10.3390/molecules27238633/s1, Figure S1: Evaluation of the cytotoxic effect of alkyltriazoles and alkylphospholipid compounds on leukemic lineage cells for 24 h; Figure S2: Evaluation of the cytotoxic effect of alkyltriazoles and alkylphospholipid compounds on leukemic cell lines after 48 h; Figure S3: Concentration–response curve of cytotoxicity of compounds **9**, **21** and vinblastine with PBMCs.; Figure S4: Protein–ligand interactions of the compounds **C9** and **C21** and cathepsins B and L by simulation; Figure S5: Top poses selected for compounds **C9** and **C21** and cathepsins B and L. Figure S6: Averages of root-mean-square-deviation (RMSD) (**A**) and radius of gyration (**B**) of compounds and cathepsins. Table S1: Information about Alkylphospholipid and alkyltriazole compounds; Table S2: Human cell types used in the study.

## References

[1] Van Blitterswijk W.J., Verheij M. (2013). Anticancer mechanisms and clinical application of alkylphospholipids. Biochim. Biophys. Acta.

[2] Carrasco M.P., Jimenez-Lopez J.M., Segovia J.L., Marco C. (2008). Hexadecylphosphocholine interferes with the intracellular transport of cholesterol in HepG2 cells. FEBS J..

[3] Mollinedo F., Gajate C., Martin-Santamaria S., Gago F. (2004). ET-18-OCH_3_ (edelfosine): A selective antitumour lipid targeting apoptosis through intracellular activation of Fas/CD95 death receptor. Curr. Med. Chem..

[4] Busto J.V., Del Canto-Janez E., Goni F.M., Mollinedo F., Alonso A. (2008). Combination of the anti-tumour cell ether lipid edelfosine with sterols abolishes haemolytic side effects of the drug. J. Chem. Biol..

[5] Mollinedo F., de la Iglesia-Vicente J., Gajate C., Estella-Hermoso de Mendoza A., Villa-Pulgarin J.A., de Frias M., Roue G., Gil J., Colomer D., Campanero M.A. (2010). In Vitro and In Vivo selective antitumor activity of Edelfosine against mantle cell lymphoma and chronic lymphocytic leukemia involving lipid rafts. Clin. Cancer Res. An. Off. J. Am. Assoc. Cancer Res..

[6] Gajate C., Matos-da-Silva M., Dakir el H., Fonteriz R.I., Alvarez J., Mollinedo F. (2012). Antitumor alkyl-lysophospholipid analog edelfosine induces apoptosis in pancreatic cancer by targeting endoplasmic reticulum. Oncogene.

[7] Hac-Wydro K., Dynarowicz-Latka P. (2010). Effect of edelfosine on tumor and normal cells model membranes—A comparative study. Colloids Surf. B Biointerfaces.

[8] Khademvatan S., Gharavi M.J., Rahim F., Saki J. (2011). Miltefosine-induced apoptotic cell death on Leishmania major and *L. tropica* strains. Korean J. Parasitol..

[9] Nieto-Miguel T., Gajate C., Mollinedo F. (2006). Differential targets and subcellular localization of antitumor alkyl-lysophospholipid in leukemic versus solid tumor cells. J. Biol. Chem..

[10] Gajate C., Del Canto-Janez E., Acuna A.U., Amat-Guerri F., Geijo E., Santos-Beneit A.M., Veldman R.J., Mollinedo F. (2004). Intracellular triggering of Fas aggregation and recruitment of apoptotic molecules into Fas-enriched rafts in selective tumor cell apoptosis. J. Exp. Med..

[11] Dorlo T.P., Balasegaram M., Beijnen J.H., de Vries P.J. (2012). Miltefosine: A review of its pharmacology and therapeutic efficacy in the treatment of leishmaniasis. J. Antimicrob. Chemother..

[12] Becher O.J., Millard N.E., Modak S., Kushner B.H., Haque S., Spasojevic I., Trippett T.M., Gilheeney S.W., Khakoo Y., Lyden D.C. (2017). A phase I study of single-agent perifosine for recurrent or refractory pediatric CNS and solid tumors. PLoS ONE.

[13] Hasegawa K., Kagabu M., Mizuno M., Oda K., Aoki D., Mabuchi S., Kamiura S., Yamaguchi S., Aoki Y., Saito T. (2017). Phase II basket trial of perifosine monotherapy for recurrent gynecologic cancer with or without PIK3CA mutations. Invest. New Drugs.

[14] Bagley R.G., Kurtzberg L., Rouleau C., Yao M., Teicher B.A. (2011). Erufosine, an alkylphosphocholine, with differential toxicity to human cancer cells and bone marrow cells. Cancer Chemother. Pharmacol..

[15] Rios-Marco P., Marco C., Galvez X., Jimenez-Lopez J.M., Carrasco M.P. (2017). Alkylphospholipids: An update on molecular mechanisms and clinical relevance. Biochim. Biophys. Acta Biomembr..

[16] Pachioni Jde A., Magalhaes J.G., Lima E.J., Bueno Lde M., Barbosa J.F., de Sa M.M., Rangel-Yagui C.O. (2013). Alkylphospholipids—A promising class of chemotherapeutic agents with a broad pharmacological spectrum. J. Pharm. Pharm. Sci..

[17] Gontijo V.S., Oliveira M.E., Resende R.J., Fonseca A.L., Nunes R.R., Comar M., Taranto A.G., Torres N.M.P.O., Viana G.H.R., Silva L.M. (2015). Long-chain alkyltriazoles as antitumor agents: Synthesis, physicochemical properties, and biological and computational evaluation. Med. Chem Res..

[18] Gontijo V.S., Espuri P.F., Alves R.B., de Camargos L.F., Santos F.V., de Souza Judice W.A., Marques M.J., Freitas R.P. (2015). Leishmanicidal, antiproteolytic, and mutagenic evaluation of alkyltriazoles and alkylphosphocholines. Eur. J. Med. Chem..

[19] Xu Z. (2020). 1,2,3-Triazole-containing hybrids with potential antibacterial activity against methicillin-resistant Staphylococcus aureus (MRSA). Eur. J. Med. Chem..

[20] Slavova K.I., Todorov L.T., Belskaya N.P., Palafox M.A., Kostova I.P. (2020). Developments in the Application of 1,2,3-Triazoles in Cancer Treatment. Recent Pat. Anti-Cancer Drug Discov..

[21] Huang Q., Xie L., Chen X., Yu H., Lv Y., Huang X., Ying J., Zheng C., Cheng Y., Huang J. (2018). Synthesis and anticancer activity of novel rapamycin C-28 containing triazole moiety compounds. Arch. Der Pharm..

[22] Alam M.M. (2022). 1,2,3-Triazole hybrids as anticancer agents: A review. Arch. Der Pharm..

[23] Kumar S., Saha S.T., Gu L., Palma G., Perumal S., Singh-Pillay A., Singh P., Anand A., Kaur M., Kumar V. (2018). 1H-1,2,3-Triazole Tethered Nitroimidazole-Isatin Conjugates: Synthesis, Docking, and Anti-Proliferative Evaluation against Breast Cancer. ACS Omega.

[24] Sambasiva Rao P., Kurumurthy C., Veeraswamy B., Santhosh Kumar G., Shanthan Rao P., Pamanji R., Venkateswara Rao J., Narsaiah B. (2013). Synthesis of novel 2-alkyl triazole-3-alkyl substituted quinoline derivatives and their cytotoxic activity. Bioorganic Med. Chem. Lett..

[25] Yang Y.L., Xiang Z.J., Yang J.H., Wang W.J., Xu Z.C., Xiang R.L. (2021). Adverse Effects Associated with Currently Commonly Used Antifungal Agents: A Network Meta-Analysis and Systematic Review. Front. Pharm..

[26] Franklim T.N., Freire-de-Lima L., Chaves O.A., LaRocque-de-Freitas I.F., Silva-Trindade J.D.d., Netto-Ferreira J.C., Freire-de-Lima C.G., Decoté-Ricardo D., Previato J.O., Mendonça-Previato L. (2019). Design, Synthesis, Trypanocidal Activity, and Studies on Human Album in Interaction of Novel S-Alkyl-1,2,4-triazoles %. J. Braz. Chem. Soc..

[27] NIH-NCI, Advances in Leukemia Research. U.S. Department of Health and Human Services, National Institutes of Health. National Cancer Institute. 2021. https://www.cancer.gov/types/leukemia/research.

[28] Portell C.A., Advani A.S. (2014). Novel targeted therapies in acute lymphoblastic leukemia. Leuk. Lymphoma.

[29] Lopez-Otin C., Matrisian L.M. (2007). Emerging roles of proteases in tumour suppression. Nat. Rev. Cancer.

[30] Pandey G., Bakhshi S., Thakur B., Jain P., Chauhan S.S. (2018). Prognostic significance of cathepsin L expression in pediatric acute myeloid leukemia. Leuk. Lymphoma.

[31] Pandey G., Bakhshi S., Kumar M., Thakur B., Jain P., Kaur P., Chauhan S.S. (2019). Prognostic and therapeutic relevance of cathepsin B in pediatric acute myeloid leukemia. Am. J. Cancer Res..

[32] Peng S., Yang Q., Li H., Pan Y., Wang J., Hu P., Zhang N. (2021). CTSB Knockdown Inhibits Proliferation and Tumorigenesis in HL-60 Cells. Int. J. Med. Sci..

[33] Samaiya M., Bakhshi S., Shukla A.A., Kumar L., Chauhan S.S. (2011). Epigenetic regulation of cathepsin L expression in chronic myeloid leukaemia. J. Cell Mol. Med..

[34] De Duve C. (2005). The lysosome turns fifty. Nat. Cell Biol..

[35] Repnik U., Stoka V., Turk V., Turk B. (2012). Lysosomes and lysosomal cathepsins in cell death. Biochim. Biophys. Acta.

[36] Stubbs M.T., Laber B., Bode W., Huber R., Jerala R., Lenarcic B., Turk V. (1990). The Refined 2.4a X-Ray Crystal-Structure of Recombinant Human Stefin-B in Complex with the Cysteine Proteinase Papain—A Novel Type of Proteinase-Inhibitor Interaction. EMBO J..

[37] Peters O.M., Ghasemi M., Brown R.H. (2015). Emerging mechanisms of molecular pathology in ALS. J. Clin. Investig..

[38] Hojjat-Farsangi M. (2016). Targeting non-receptor tyrosine kinases using small molecule inhibitors: An overview of recent advances. J. Drug Target..

[39] Van Blitterswijk W.J., Verheij M. (2008). Anticancer alkylphospholipids: Mechanisms of action, cellular sensitivity and resistance, and clinical prospects. Curr. Pharm. Des..

[40] Yosifov D.Y., Kaloyanov K.A., Guenova M.L., Prisadashka K., Balabanova M.B., Berger M.R., Konstantinov S.M. (2014). Alkylphosphocholines and curcumin induce programmed cell death in cutaneous T-cell lymphoma cell lines. Leuk. Res..

[41] Leonard R., Hardy J., van Tienhoven G., Houston S., Simmonds P., David M., Mansi J. (2001). Randomized, double-blind, placebo-controlled, multicenter trial of 6% miltefosine solution, a topical chemotherapy in cutaneous metastases from breast cancer. J. Clin. Oncol. Off. J. Am. Soc. Clin. Oncol..

[42] Chakrabandhu K., Huault S., Hueber A.O. (2008). Distinctive molecular signaling in triple-negative breast cancer cell death triggered by hexadecylphosphocholine (miltefosine). FEBS Lett..

[43] Chiarini F., Del Sole M., Mongiorgi S., Gaboardi G.C., Cappellini A., Mantovani I., Follo M.Y., McCubrey J.A., Martelli A.M. (2008). The novel Akt inhibitor, perifosine, induces caspase-dependent apoptosis and downregulates P-glycoprotein expression in multidrug-resistant human T-acute leukemia cells by a JNK-dependent mechanism. Leukemia.

[44] Vital W.D., Torquato H.F.V., Jesus L.O.P., Judice W.A.S., Silva M., Rodrigues T., Justo G.Z., Veiga T.A.M., Paredes-Gamero E.J. (2019). 4-Deoxyraputindole C induces cell death and cell cycle arrest in tumor cell lines. J. Cell. Biochem..

[45] Paredes-Gamero E.J., Casaes-Rodrigues R.L., Moura G.E., Domingues T.M., Buri M.V., Ferreira V.H., Trindade E.S., Moreno-Ortega A.J., Cano-Abad M.F., Nader H.B. (2012). Cell-permeable gomesin peptide promotes cell death by intracellular Ca^2+^ overload. Mol. Pharm..

[46] Buri M.V., Domingues T.M., Paredes-Gamero E.J., Casaes-Rodrigues R.L., Rodrigues E.G., Miranda A. (2013). Resistance to degradation and cellular distribution are important features for the antitumor activity of gomesin. PLoS ONE.

[47] De Ford C., Ulloa J.L., Catalan C.A.N., Grau A., Martino V.S., Muschietti L.V., Merfort I. (2015). The sesquiterpene lactone polymatin B from Smallanthus sonchifolius induces different cell death mechanisms in three cancer cell lines. Phytochemistry.

[48] Vieira Torquato H.F., Ribeiro-Filho A.C., Buri M.V., Araujo Junior R.T., Pimenta R., de Oliveira J.S., Filho V.C., Macho A., Paredes-Gamero E.J., de Oliveira Martins D.T. (2017). Canthin-6-one induces cell death, cell cycle arrest and differentiation in human myeloid leukemia cells. Biochim. Biophys. Acta Gen. Subj..

[49] Torquato H.F.V., Junior M.T.R., Lima C.S., Junior R.T.A., Talhati F., Dias D.A., Justo G.Z., Ferreira A.T., Pilli R.A., Paredes-Gamero E.J. (2022). A canthin-6-one derivative induces cell death by apoptosis/necroptosis-like with DNA damage in acute myeloid cells. Biomed. Pharm..

[50] Wang Q., Li Y.Y., Cheng J.Y., Chen L., Xu H., Li Q.H., Pang T.X. (2016). Sam68 affects cell proliferation and apoptosis of human adult T-acute lymphoblastic leukemia cells via AKT/mTOR signal pathway. Leuk. Res..

[51] Mu Q.T., Ma Q.L., Lu S.S., Zhan T., Yu M.X., Huang X., Chen J., Jin J. (2014). 10058-F4, a c-Myc inhibitor, markedly increases valproic acid-induced cell death in Jurkat and CCRF-CEM T-lymphoblastic leukemia cells. Oncol. Lett..

[52] Van Vlierberghe P., Ferrando A. (2012). The molecular basis of T cell acute lymphoblastic leukemia. J. Clin. Investig..

[53] Chiaretti S., Foa R. (2009). T-cell acute lymphoblastic leukemia. Haematol-Hematol J..

[54] Girardi T., Vicente C., Cools J., De Keersmaecker K. (2017). The genetics and molecular biology of T-ALL. Blood.

[55] Graf M., Reif S., Hecht K., Pelka-Fleischer R., Pfister K., Schmetzer H. (2005). High expression of urokinase plasminogen activator receptor (UPA-R) in acute myeloid leukemia (AML) is associated with worse prognosis. Am. J. Hematol..

[56] Jain M., Bakhshi S., Shukla A.A., Chauhan S.S. (2010). Cathepsins B and L in peripheral blood mononuclear cells of pediatric acute myeloid leukemia: Potential poor prognostic markers. Ann. Hematol..

[57] Gocheva V., Zeng W., Ke D.X., Klimstra D., Reinheckel T., Peters C., Hanahan D., Joyce J.A. (2006). Distinct roles for cysteine cathepsin genes in multistage tumorigenesis. Genes Dev..

[58] Leatherbarrow R.J. (2010). GraFit, Version 5.0.13.

[59] Copeland R.A. (2005). Evaluation of enzyme inhibitors in drug discovery. A guide for medicinal chemists and pharmacologists. Methods Biochem. Anal..

[60] Morris G.M., Goodsell D.S., Halliday R.S., Huey R., Hart W.E., Belew R.K., Olson A.J. (1998). Automated docking using a Lamarckian genetic algorithm and an empirical binding free energy function. J. Comput. Chem..

[61] Liu Y., Grimm M., Dai W.T., Hou M.C., Xiao Z.X., Cao Y. (2020). CB-Dock: A web server for cavity detection-guided protein-ligand blind docking. Acta Pharm. Sin..

[62] Zhang W.Y., Bell E.W., Yin M.H., Zhang Y. (2020). EDock: Blind protein-ligand docking by replica-exchange monte carlo simulation. J. Cheminform..

[63] Trott O., Olson A.J. (2010). Software News and Update AutoDock Vina: Improving the Speed and Accuracy of Docking with a New Scoring Function, Efficient Optimization, and Multithreading. J. Comput. Chem..

[64] DeLano W.L. (2000). The PyMOL Molecular Graphics System.

[65] Humphrey W., Dalke A., Schulten K. (1996). VMD: Visual molecular dynamics. J. Mol. Graph. Model..

[66] Wang J.M., Wolf R.M., Caldwell J.W., Kollman P.A., Case D.A. (2004). Development and testing of a general amber force field. J. Comput. Chem..

[67] Jakalian A., Bush B.L., Jack D.B., Bayly C.I. (2000). Fast, efficient generation of high-quality atomic Charges. AM1-BCC model: I. Method. J. Comput. Chem..

[68] Maier J.A., Martinez C., Kasavajhala K., Wickstrom L., Hauser K.E., Simmerling C. (2015). ff14SB: Improving the Accuracy of Protein Side Chain and Backbone Parameters from ff99SB. J. Chem. Theory Comput..

[69] Jorgensen W.L., Chandrasekhar J., Madura J.D., Impey R.W., Klein M.L. (1983). Comparison of Simple Potential Functions for Simulating Liquid Water. J. Chem. Phys..

[70] Case D.A.B.R.M., Cerutti D.S., Cheatham T.E., Darden T.A., Duke R.E., Giese T.J., Gohlke H., Goetz A.W., Homeyer N. (2016). Amber 16.

[71] Gotz A.W., Williamson M.J., Xu D., Poole D., Le Grand S., Walker R.C. (2012). Routine Microsecond Molecular Dynamics Simulations with AMBER on GPUs. 1. Generalized Born. J. Chem. Theory Comput..

[72] Salomon-Ferrer R., Gotz A.W., Poole D., Le Grand S., Walker R.C. (2013). Routine Microsecond Molecular Dynamics Simulations with AMBER on GPUs. 2. Explicit Solvent Particle Mesh Ewald. J. Chem. Theory Comput..

[73] Loncharich R.J., Brooks B.R., Pastor R.W. (1992). Langevin Dynamics of Peptides - the Frictional Dependence of Isomerization Rates of N-Acetylalanyl-N’-Methylamide. Biopolymers.

[74] Izaguirre J.A., Catarello D.P., Wozniak J.M., Skeel R.D. (2001). Langevin stabilization of molecular dynamics. J. Chem. Phys..

[75] Darden T., York D., Pedersen L. (1993). Particle Mesh Ewald: An N.Log (N) method for Ewald Sums in large systems. J. Chem. Phys..

[76] Essmann U.P.L., Berkowitz M.L. (1995). A smooth particle mesh Ewald method. J. Chem. Phys..

[77] Ryckaert J.P.C.G., Berendsen H.J.C. (1977). Numerical integration of the cartesian equations of motion of a system with constraints: Molecular dynamics of N-alkanes. J. Comput. Phys..

[78] Genheden S.K.O., Mikulskis P., Hoffmann D., Ryde U. (2012). The normal-mode entropy in the MM/GBSA method: Effect of system truncation, buffer region, and dielectric constant. J. Chem. Inf. Model..

