# Supplementary material for: In Vitro Study of Cytotoxic Mechanisms of Alkylphospholipids and Alkyltriazoles in Acute Lymphoblastic Leukemia Models
Source: Molecules. 2022 Dec 6;27(23):8633. doi: 10.3390/molecules27238633 (PMC9737184; doi:10.3390/molecules27238633)
Supplement: Supplementary file 1 [file molecules-27-08633-s001.zip › molecules-1856628-supplementary.pdf]

SUPPLEMENTARY MATERIALS

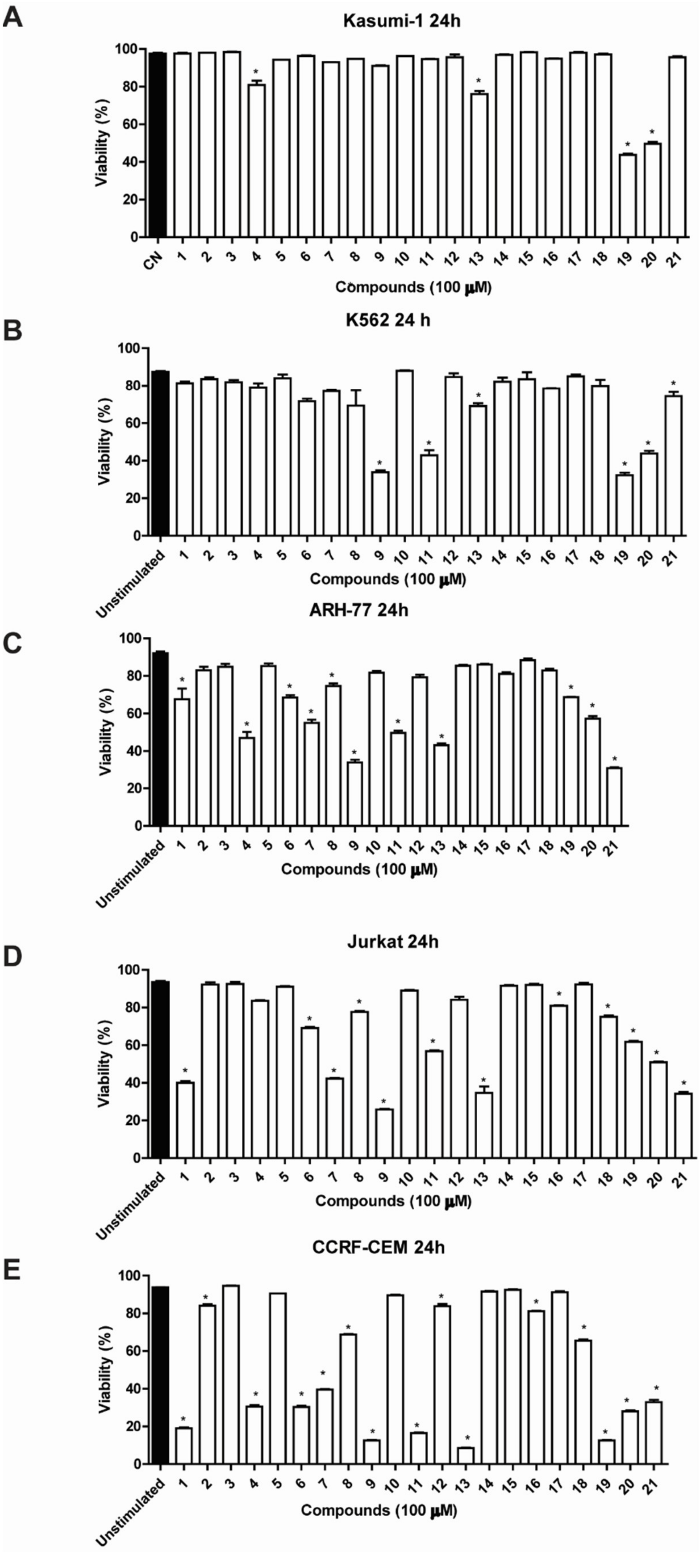

Figure S1. Evaluation of the cytotoxic effect of alkyltriazoles and alkylphospholipid compounds on leukemic lineage cells for 24 h. Cells were plated at 2x10^5 cells/mL: Kasumi-1 (A), K562 (B), ARH-77

(C), Jurkat (D) and CCRF-CEM (E), and treated with 100 μM of each compound for 24 h. Subsequently, the samples were labeled for 20 min with calcein/EthD-1. Analysis was performed by flow cytometry. The results were expressed as percentage of cell viability. The data are the mean ± SEM of 3 experiments performed in duplicate. * $p < 0.05$, relative to Unstimulated sample.

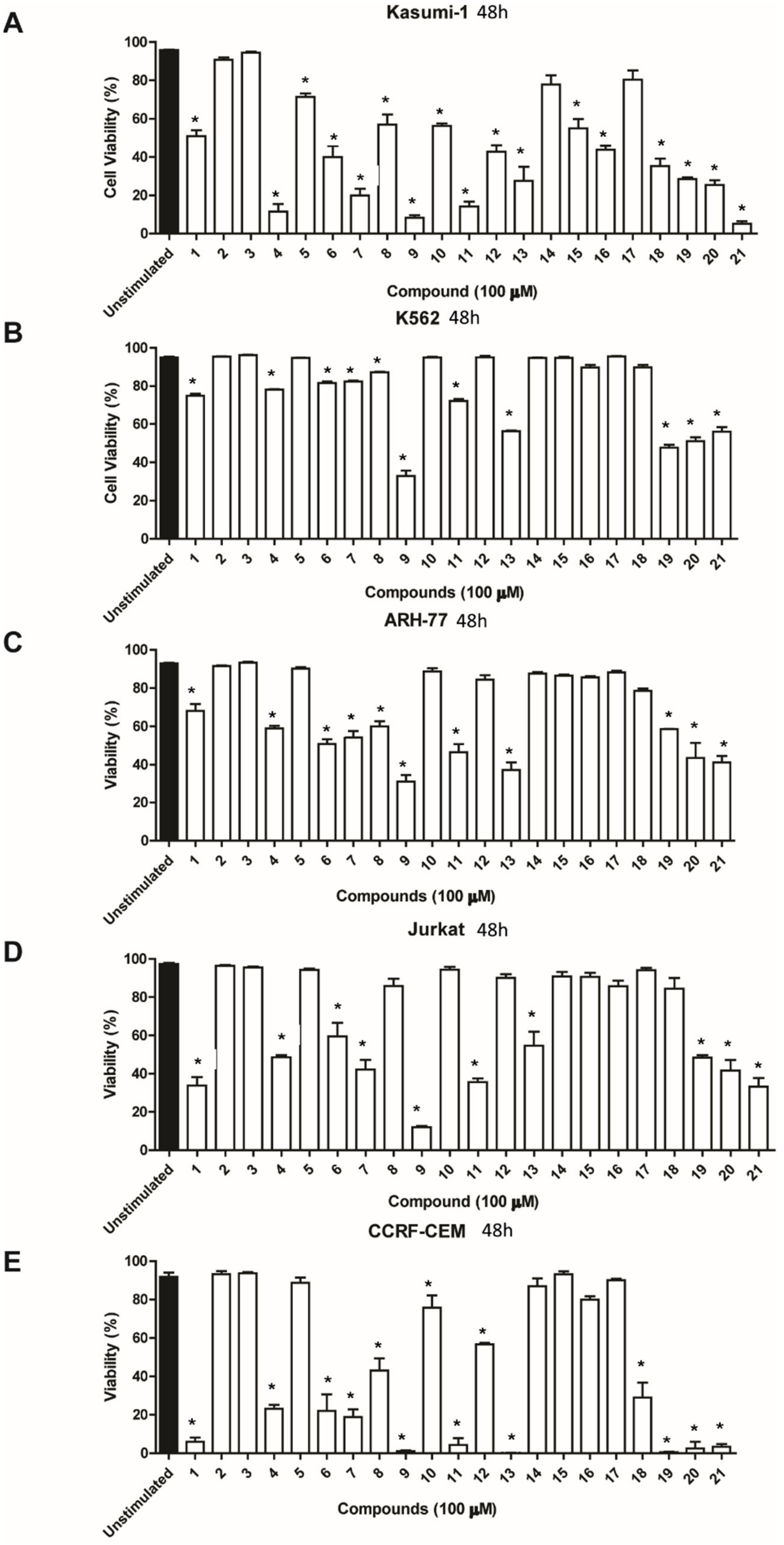

Figure S2. Evaluation of the cytotoxic effect of alkyltriazoles and alkylphospholipid compounds on leukemic cell lines for 48 h. Cells were plated at $2x10^5$ cells/mL of: Kasumi-1 (A), K562 (B), ARH-77

(C), Jurkat (D) and CCRF-CEM (E), and treated with 100 μM of each compound for 48 h. Subsequently, the samples were labeled for 20 min with calcein/EthD-1. Analysis was performed by flow cytometry. The results were expressed as percentage of cell viability. The data are the mean ± SEM of 3 experiments performed in duplicate. * $p < 0.05$, relative to Unstimulated sample.

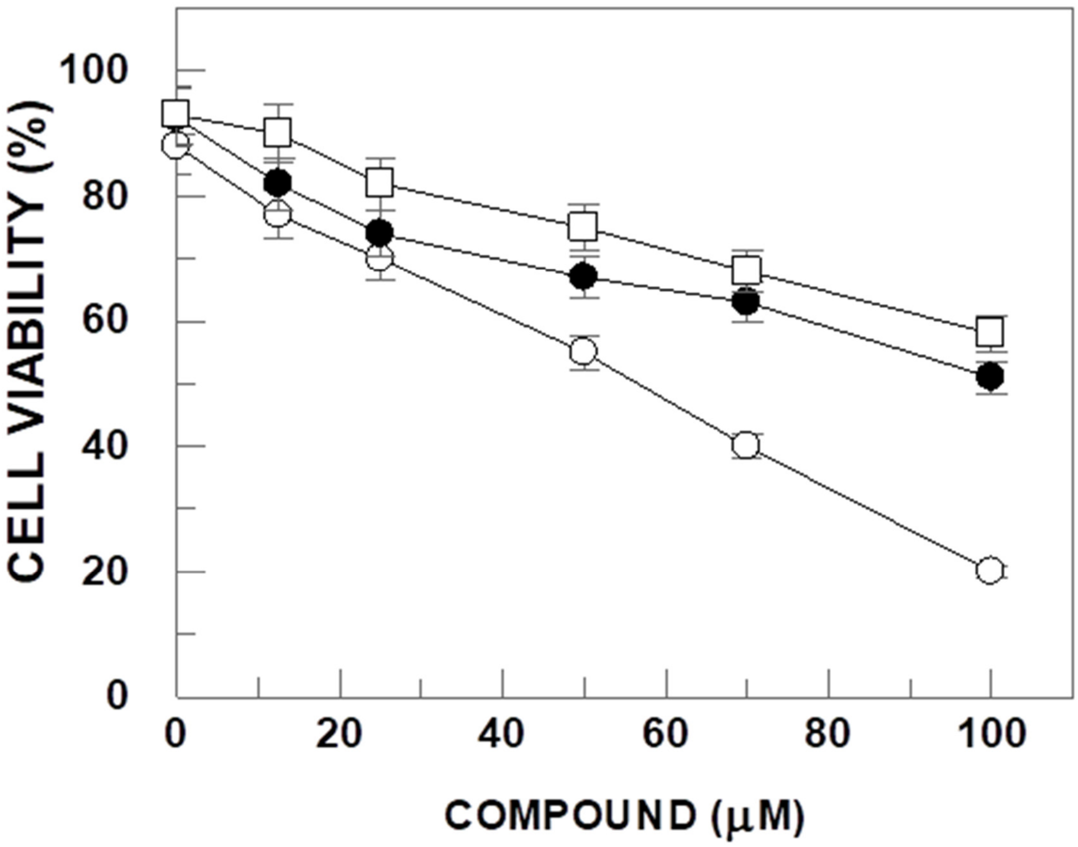

Figure S3. Concentration-response curve of cytotoxicity of compounds **C9**, **C21** and vinblastine on PBMCs. PBMCs cells were cultured (2x10^5 cells/mL) on 96-well microplates. The cells were treated with compounds **C9** (●) and **C21** (□), and vinblastine (○) (Sigma-Aldrich, Germany) for 24 h. After, the cells were labeled with Annexin V-FITC and 5 μg/mL PI. The results were expressed as percentage. The data are the mean ± SEM of 3 experiments performed in duplicate.

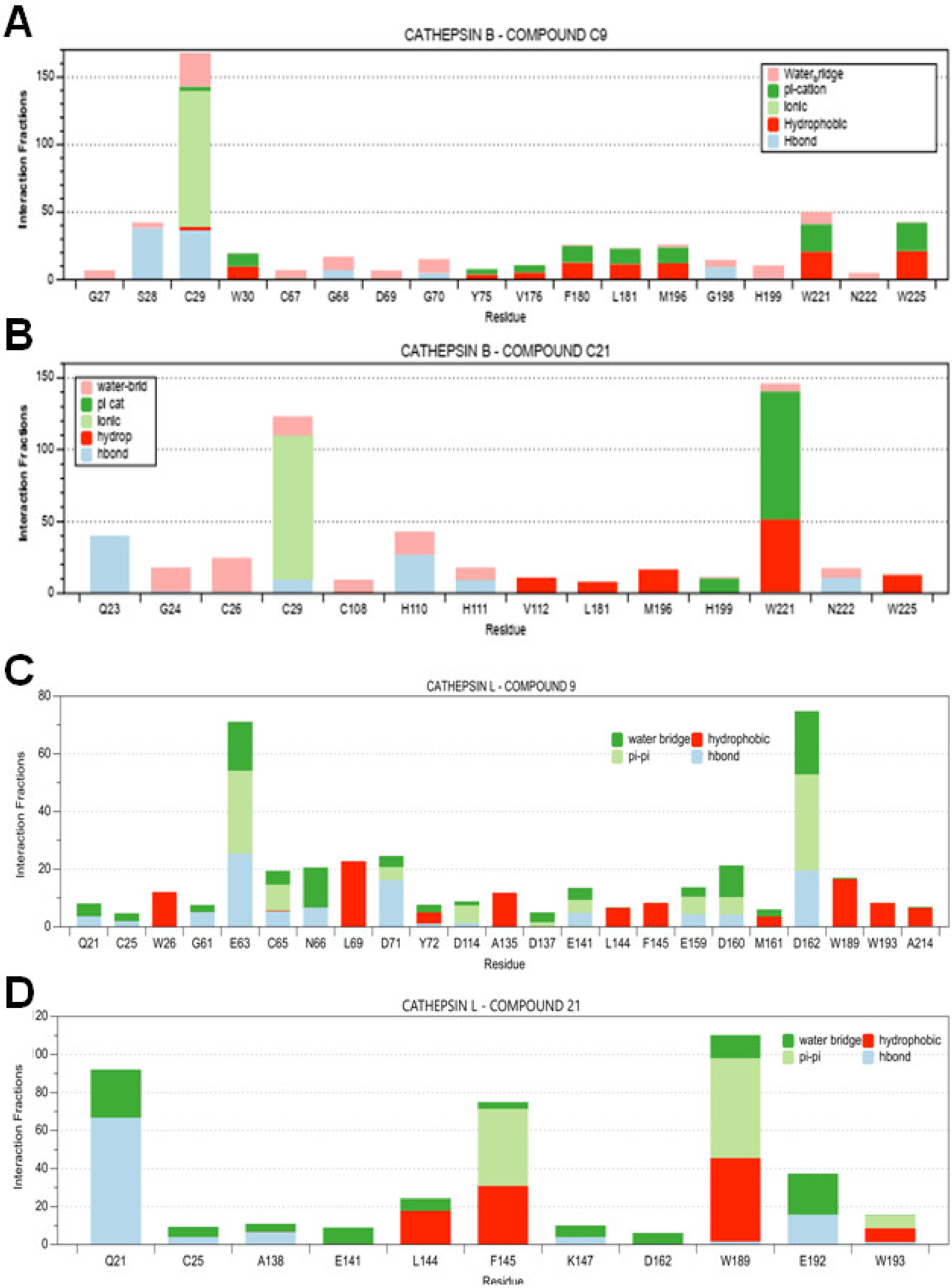

Figure S4. Protein-Ligand Contacts of the compounds **C9** and **C21** and cathepsins B and L by simulation. The stacked bar charts are normalized over the course of the trajectory of simulation. Values over 100 are possible when a protein residue make multiple contacts of same subtype with the ligand. A) Cathepsin B-**C9** complex; B) Cathepsin B-**C21** complex; C) Cathepsin L-**C9** complex; D)

Cathepsin L-**C21** complex. Salmon: water bridge: light blue: hydrogen bond; dark green: pi-cation; light green: ionic; purple: pi-pi; red: hydrophobic.

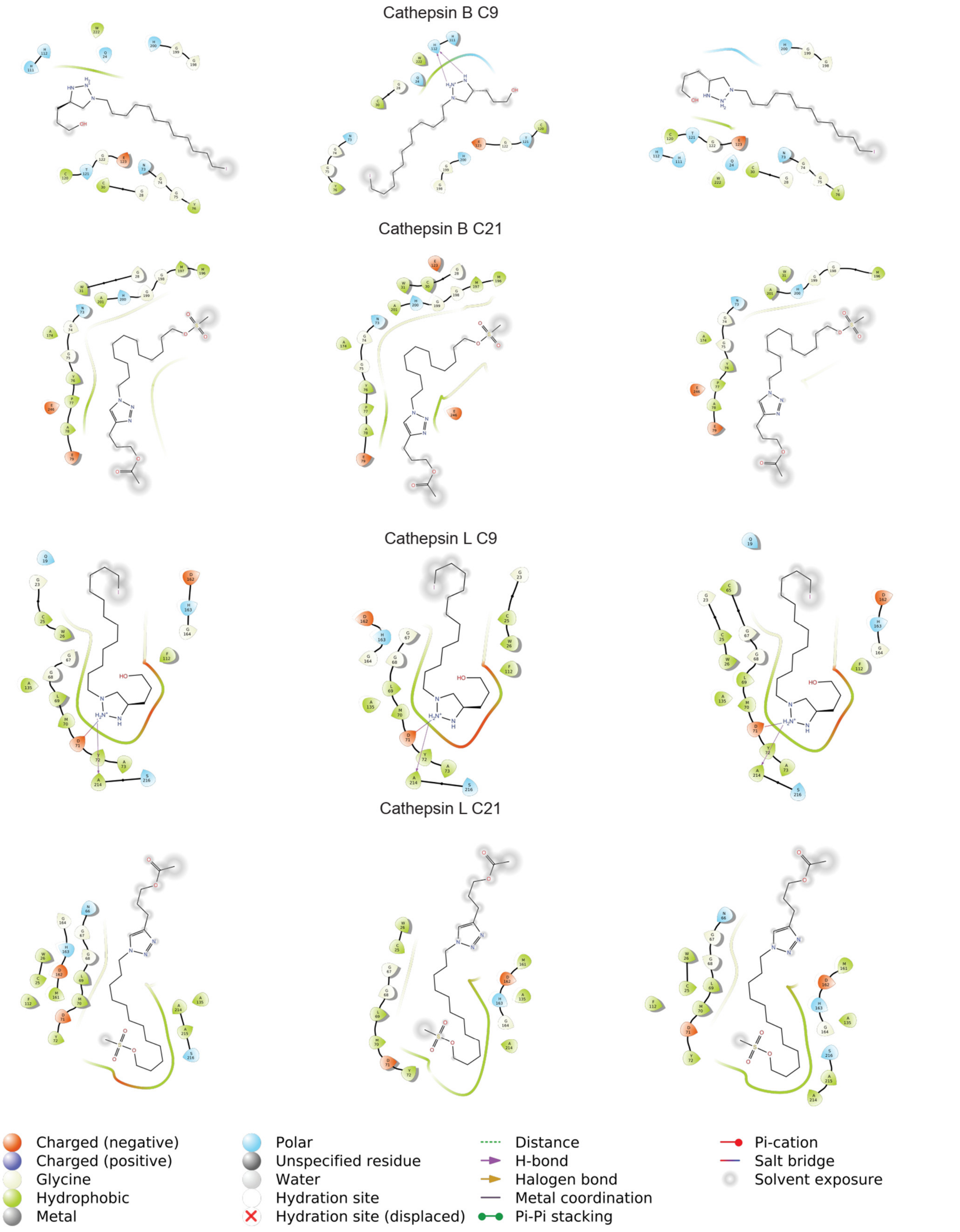

Figure S5. Top poses selected for compounds **C9** and **C21** and cathepsins B and L.

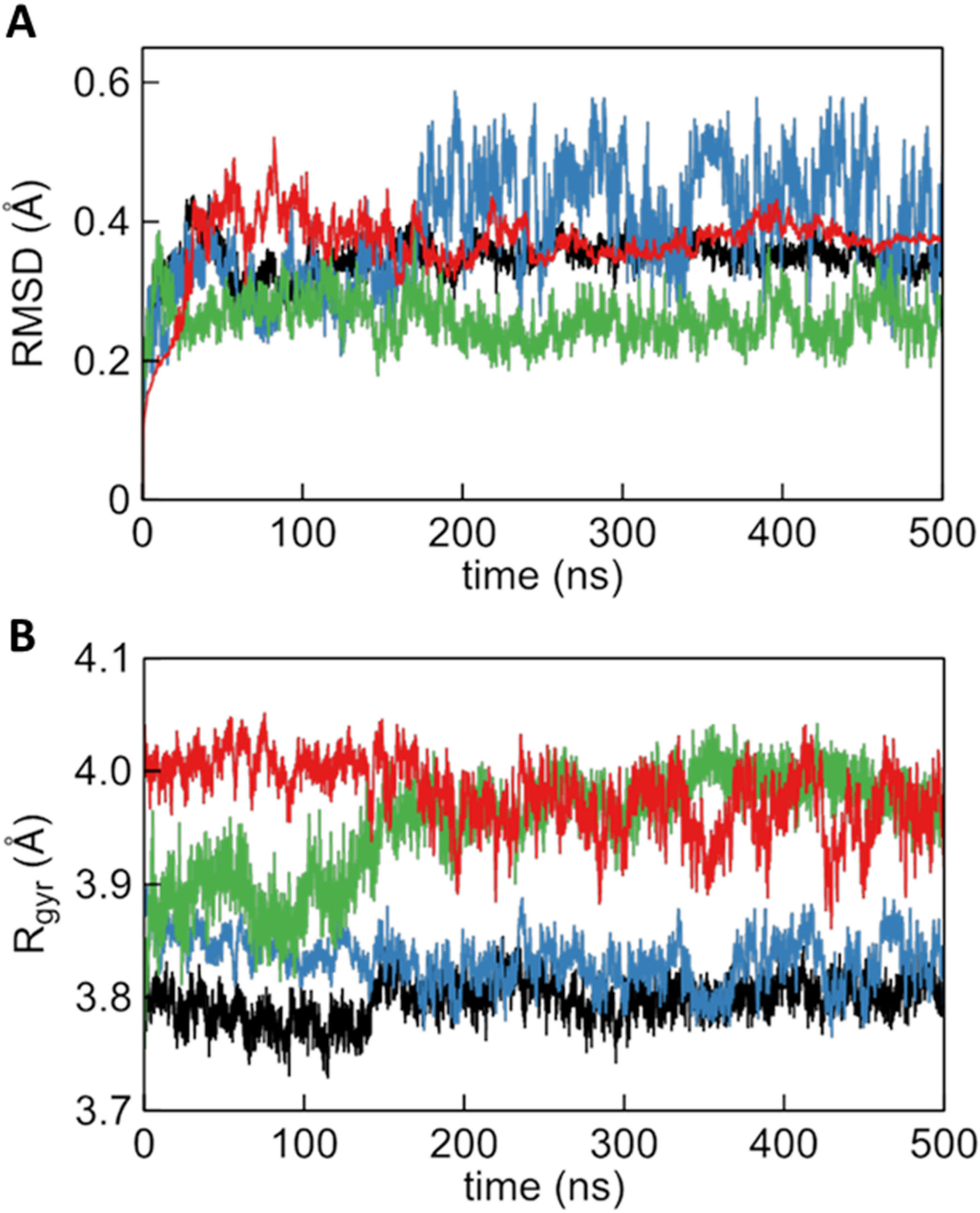

Figure S6. Averages of Root-Mean-Square-Deviation (RMSD) (A) and Radius of gyration (B) of compounds and cathepsins. Compound **C9** bound to cathepsin L (blue) and cathepsin B (green), and compound **C21** bound to cathepsin L (black) and cathepsin B (red). Average center of mass distances of **C9** was 4.2 Å for cathepsin B and 3.78 Å for cathepsin L, while **C21** center of mass distances was 4.0 Å for cathepsin B and 3.9 Å for cathepsin L.
